# Contribution of land use practices to GHGs in the Canadian Prairies crop sector

**DOI:** 10.1371/journal.pone.0260946

**Published:** 2021-12-17

**Authors:** Lana Awada, Cecil Nagy, Peter W. B. Phillips

**Affiliations:** 1 Senior Policy Fellow in Food Security, Centre for the Study of Science and Innovation Policy (CSIP), Johnson Shoyama School of Public Policy (JSGS), University of Saskatchewan, Saskatoon, Canada; 2 Research Fellow, Department Agriculture and Resource Economics, University of Saskatchewan, Saskatoon, Saskatoon, Canada; 3 Director, Centre for the Study of Science and Innovation Policy (CSIP); and Distinguished Professor, Johnson Shoyama Graduate School of Public Policy (JSGS), University of Saskatchewan, Saskatoon, Canada; Shenzhen University, CHINA

## Abstract

The global crop sector is estimated to contribute about 10.4% of global GHGs annually. The Canadian crop sector is assessed as adding about 6.5% to total national emissions. These estimates over report the impact of farming as they ignore the complex interaction of cropping with the environment and the role land use, land use change and forestry (LULUCF) play in sequestering carbon. This study quantifies the contribution of land use to GHG emissions and removals in the Canadian Prairies crop sector between 1985 and 2016. The modeling effort explores how different farming practices (i.e., conventional tillage (CT), minimum tillage (MT), zero tillage (ZT), summerfallow, crop rotations, and residue retention) and input usage rates (i.e., fertilizer and fuel) affect GHG emissions in different soil climate zones and provinces in the Prairies region. The adoption of sustainable practices led to an 80% decline in GHG emissions in the crop sector between 1985 and 2016. Since 2005, the baseline for Canada’s Paris commitment, sectoral emissions dropped 53%, more than is required to meet the 2030 target. Most promising, the crop sector was a net GHG sink between 2013 and 2016 in Alberta and between 2006 and 2016 in Saskatchewan. As positive as these developments have been, more can be done by directing research to identify options for reducing GHGs in Manitoba (which made only minimal improvements as farmers there faced conditions requiring continuous use of conventional tillage practices), to explore better nitrogen management (a major continuing source of GHG from cropping) and by searching for low carbon transport options.

## Introduction

Canada ranks as the fourth largest per capita emitter of GHGs among Organization for Economic Co-operation and Development (OECD) countries, accounting, in aggregate, for about 2% of global emissions in 2019. In 2016, Canada’s emission intensity was 0.49 kg CO_2_-equivalents (CO_2_eq), higher than the OECD average of 0.34 kg CO_2_eq [1]. By 2016, emissions intensity decreased by 35% from 1990 and 19% from 2005, reflecting that Canada’s economy has grown much faster than its GHG emissions [1]. Although, Canada’s per capita emissions decreased from 22.7 tonne (t) of CO_2_eq in 2005 to 19.4 t CO_2_eq in 2016, this remains higher than the OECD average of 12.4 t CO_2_eq [2]. In 2016, total annual emissions in Canada reached 704 million tonnes (Mt) of CO_2_eq, only 3.8% below the 2005 level of emissions. The level of emissions has decreased in all Canada’s provinces and territories since 2005, except in Alberta (AB) (+14%), Saskatchewan (SK) (+10%) and Manitoba (MB) (+3.5%) [2].

In response, Canada has signed on to all the major climate change agreements: The United Nations Conference on Environment and Development (the Earth Summit) (1992); The Kyoto Protocol (2002); the Copenhagen Accord (2009); and the Paris Agreement (2016). The current global goal is to keep the temperature rise this century to well below 2 degrees Celsius above pre-industrial levels and to pursue efforts to limit the temperature increase to below 1.5 degrees Celsius. Each country has made “Nationally Determined Contributions” (NDC) in response to the global targets.

Canada’s NDCs are reflected in the 2016 Pan-Canadian Framework (PCF) on Clean Growth and Climate Change, which is in its implementation stage. The objective of the PCF is to simultaneously mitigate emissions, increase resilience and ensure low-carbon economic growth. The framework builds on commitments and actions undertaken by Canada following the 21^st^ Conference of the Parties (COP21) to the United Nations Framework Convention on Climate Change (UNFCCC) in Paris. In COP21, Canada committed to reduce GHG emissions by 30 percent below 2005 levels by 2030 [2]; in 2021 the federal government announced that it would enhance Canada’s NDCs to 40–45% below 2005 levels by 2030. Canada has yet to fully develop and implement policies that would meet its emission reduction commitments.

In 2016, the agriculture sector contributed about 16.2% of global GHG emissions, with about 10.4% from cropping activities and 5.8% from livestock production. Canadian agriculture is more efficient, contributing only about 10% of total Canada’s emissions, not adjusting for GHG emission removals by land use, land use change and forestry (LULUCF) [2]. Using the international ratio of crops to animal production, about 6.5% of Canada’s emissions are assigned to crop-based agriculture. Agricultural sector emissions are estimated to have increased by 24% between 1990 and 2016, but that does not make any adjustment for the fact that land can and often does act as a carbon ‘sink’ for atmospheric GHG.

The Pan Canadian Framework is working to take into account the GHG emissions and removals by LULUCF in the national inventory of GHGs [3]. Much of the analysis is rather aggregate level (combined crop and livestock sectors) or not fully representing the changes in land use practices. Depending on crop production practices, LULUCF can lower or increase the net GHG emissions for agriculture. This is particularly important for the Canadian Prairies’ provinces—Alberta, Saskatchewan, and Manitoba—as they account for approximately 82% of Canada’s farmland or about 52 Mha (20 Mha in Alberta, 25 Mha in Saskatchewan and 7 Mha in Manitoba Mha) [4].

The objective of this study is to fill this gap by quantifying the contribution of land use to GHG emissions and removals in the Canadian Prairies crop sector between 1985 and 2016. This includes assessing the emissions and sinks associated with different crop production practices (i.e., conventional tillage (CT), minimum tillage (MT), zero tillage (ZT), summerfallow, crop rotations, and residue retention) and rates of input usage (i.e., fertilizer and fuel), adjusted to reflect the type of crops and soil climate zones in the Prairies region. The study measures the main GHGs in crop production, including: (1) soil carbon stock (SCS) as a net source of or sink for CO_2_; (2) N_2_O emissions from fertilizer application; (3) N_2_O emissions from retained crop residue; (4) N_2_O emissions from summerfallow; and (5) CO_2_ emissions from fuel used on farm and for transportation (all converted to CO_2_eq based on GWP_100_ time horizon). The increase in soil carbon stock is key to alleviating the greenhouse effects caused by land use. This study shows that the increase in SCS due to lower tillage practices and higher crop residue retention can fully mitigate the emissions associated with fertilizer and fuel use in crop production. Our results are presented at the disaggregated level for each of the Prairie soil climate zones and the three provinces (Alberta, Saskatchewan, and Manitoba), and at the aggregated level for the Canadian Prairies region. The real dollar values of the GHGs are calculated and then discounted to estimate the net present values over the period of 1985 to 2016.

To monitor the relative comparative performance of GHG emissions and to provide policy decision-makers with relevant information about the magnitude of net GHG emissions in the crop sector, we compare estimates for 2016 with those of 1985 (the base year for the data) and 2005 (the reference year for Canada’s NDCs for the Paris Accord). The results of this study provide evidence-based measures that identify and quantify land use practices that contribute to or mitigate GHG emissions than can be used by policymakers to make more effective decisions in developing and implementing climate policies in agriculture.

This study complements the work by Awada and Nagy [5], which measured the GHG sources and sinks in the crop sector in Alberta and Manitoba. It confirms their findings that the adoption of sustainable practices is key to mitigate GHG emissions in agriculture. As an extension, this study includes the province of Saskatchewan and the aggregate Canadian Prairies region to the measurement of GHG emissions in the crop sector.

## The study area

**Fig 1:**
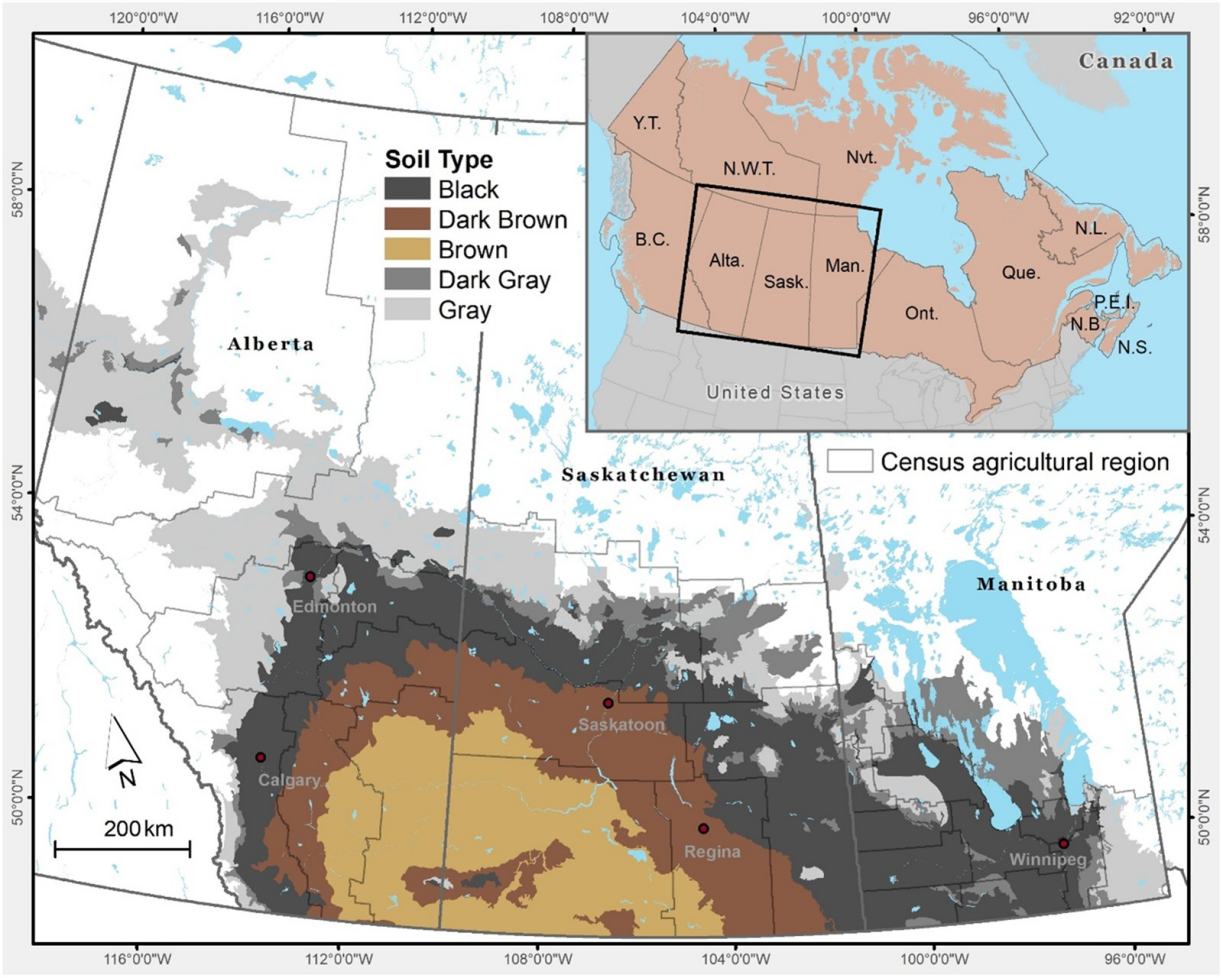
Distribution of major soil zones in the Canadian Prairies. Fig 1 is built at the University of Saskatchewan, Canada, using the Canadian Hub for Applied and Social Research, Mapping and Geographic Information Systems service (https://chasr.usask.ca/services/our_services/mapping-and-data-visualizations.php#MappingandGeographicInformationSystems). The main layers (Soil Zone and Census agriculture region) are downloaded from the following sites: (1) Soil zones from Prairie Soil Zones of Canada - Open Government Portal [6], and (2) Census agriculture region (filename: lcar000b16a_e) from Statistics Canada website (2016 Census Boundary files (statcan.gc.ca) [7]. The supporting layers are also downloaded from the Statistics Canada site (2016 Census Boundary files (statcan.gc.ca) [7]. All files are provided under the Statistics Canada Open Licence (Statistics Canada Open Licence (statcan.gc.ca) and Open Government Licence - Canada | Open Government, Government of Canada.

Our study focuses on the Canadian Prairie provinces of Alberta, Saskatchewan, and Manitoba. The Canadian Prairie provinces are bounded by latitudes 60 and 49^0^N and longitudes 120-95^0^W (Fig 1) [6, 7]. The major agricultural ecological soil zones of the Prairies are Brown, Dark-brown, Black (Thin-black and Thick-black), and Grey and Dark-grey (Fig 1). About 57% of the cropped land is located in the Black and Grey soil zones; 22% in the Dark-brown; and the remainder in the Brown soil zone [8]. The Brown soil zone is the most arid zone. Black and Grey soil zones are cooler and receive more precipitation than Brown soil zones. Annual precipitation increases from 275 mm in the Brown soil zones to 475 mm in the Black and Grey soil zones. The soil organic matter content of the surface 30 cm is about 2–5% in the Brown soil zones and characterized by relatively low to moderate soil fertility; 5–10% in the Black soil zones, indicating high levels of fertile soils; and ranges from 1 to 4% in the Grey soil zones, delivering relatively low soil fertility [9]. Mean annual temperatures are higher in the Brown soil zones (the most arid zone) than in the Black and Gray soil zones. Annual mean temperature on the Prairies ranges between 1.0^°^C and 5.0^°^C [10].

The main crops grown on the Prairies are wheat, canola, tame hay, barley, peas, oats, lentils, flaxseed, and mustard seed. In 2020, the Prairies produced about 32 Mt of wheat, 18.6 Mt of canola, 11.0 Mt of tame hay, 10.4 Mt of barley, 4.5 Mt of peas, 4.2 Mt of oats, 3.0 Mt of lentils, 0.6 Mt of flaxseed, and 0.1 Mt mustard seed [11].

**Fig 2:**
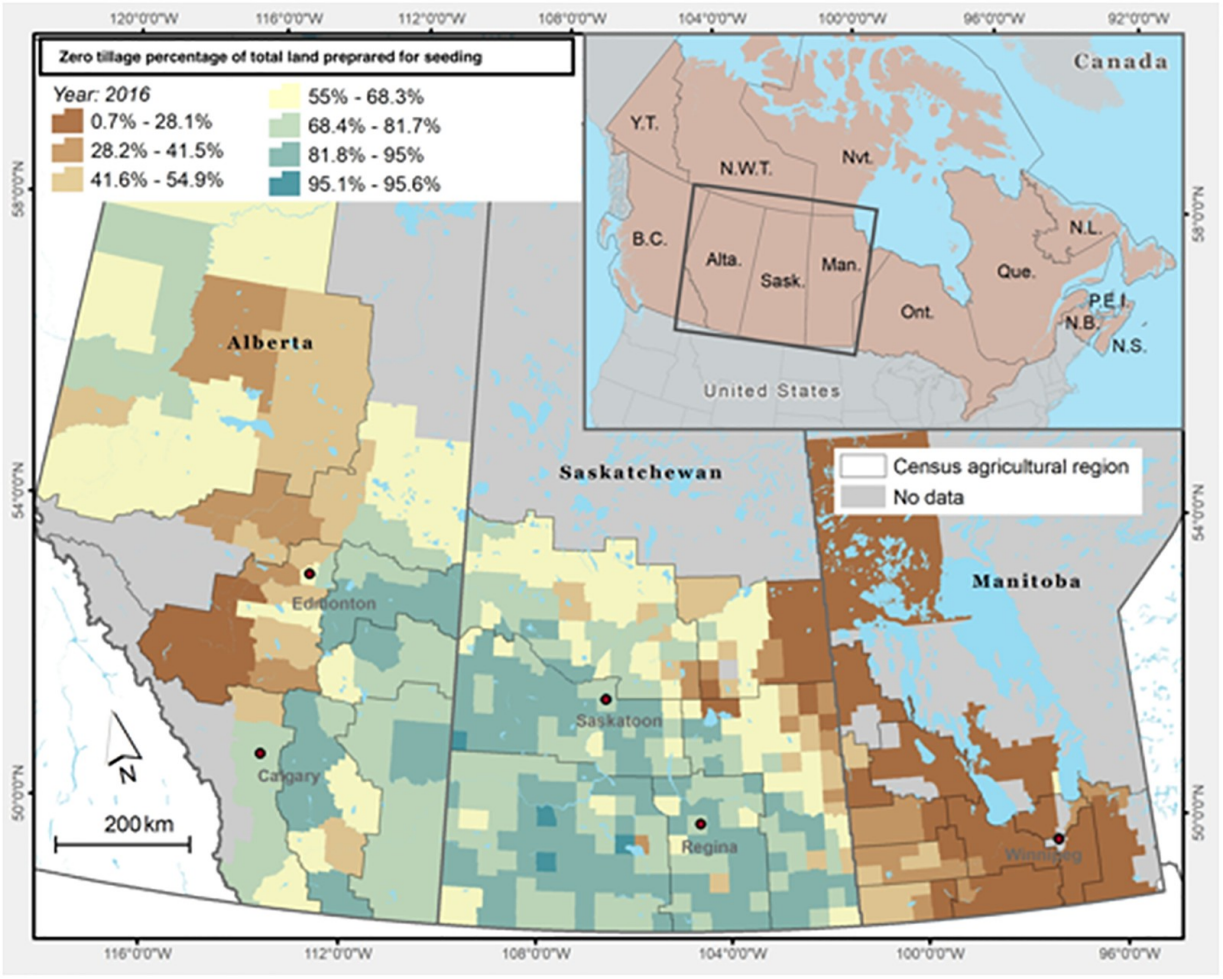
Percentage of cropland area under zero tillage (ZT) in the Canadian Prairies (2016). Fig 2 is built at the University of Saskatchewan, Canada, using the Canadian Hub for Applied and Social Research, Mapping and Geographic Information Systems service (https://chasr.usask.ca/services/our_services/mapping-and-data-visualizations.php#MappingandGeographicInformationSystems). The main layers (Soil Zone and Census agriculture region) are downloaded from the following sites: (1) Soil zones from Prairie Soil Zones of Canada - Open Government Portal [6], and (2) Census agriculture region (filename: lcar000b16a_e) from Statistics Canada website (2016 Census Boundary files (statcan.gc.ca) [7]. The supporting layers are also downloaded from the Statistics Canada site (2016 Census Boundary files (statcan.gc.ca) [7] and the Census of Agriculture Data-Land use, land tenure and management practices-Census Consolidated Subdivisions (CCS) level, Census year 2016 (https://open.canada.ca/data/en/dataset/b28888d3-69fe-47a6-a6de-94b97ff1579e [14]. All files are provided under the Statistics Canada Open Licence (Statistics Canada Open Licence (statcan.gc.ca) and Open Government Licence - Canada | Open Government, Government of Canada.

Soils that are covered by crops and crop residue have increased in the Canadian Prairies over the past four decades. Compared to bare soils, covered soils—based on the number of days in a year that arable soils are covered—are less susceptible to degradation processes (i.e., soil erosion, organic matter depletion, salinity, breakdown of soil structure and loss of fertility). Over the 1985–2011 period, the average increase in annual soil cover days in Alberta was 6.9%, in Manitoba 6.3%, and in Saskatchewan 12.4% [12]. The increase in soil cover was mainly attributed to the adoption of conservation tillage (both MT and ZT) and the decline in the frequency of summerfallow. In 2016, on average, 80% of the cropped land on the Prairies was under some form of conservation tillage, with more than 60% under ZT; only about 2% was under summerfallow [4, 13]. Fig 2 shows the percentage of cropland area under ZT in the Canadian Prairies in 2016, with Saskatchewan having the highest adoption rate at 70% [6, 7, 14]. The changes in land management practices on the Prairies contributes to the reduction of all forms of land degradation (soil erosion and salinity and organic matter depletion) and to the decrease in agricultural GHG emissions [12, 15]. The latter is the focus of this paper, in which the main GHGs in crop production is measured and discussed.

## Method and data sources

This study uses the Prairie Crop Energy Model (PCEM) as an accounting framework to quantify the annual GHG emissions and sinks [16]. In this model, crop types, inputs, soil-climate zones and cropping activities are measured. In this study, soil-climate zones are accounted for by dividing the cropland in each province into 22 crop districts—defined by Statistics Canada’s Field Crop Survey—and five soil climate zones—Brown, Dark-brown, Thin-black, Thick-black and Grey [17] (arable land in Alberta is divided into seven crop districts, Saskatchewan into nine districts and Manitoba into six districts, a description of the percentage of cropland by soil climate zones and crop districts in each province is presented in Table S1.1 in S1 Table). In each soil zone, the model allocates arable land to 122 differentiated cropping activities, defined by the type of crop grown, land management practices, crop rotations and soil characteristics. Cropping activities include the eight major grain crops (wheat, durum, feed barley, malt barley, flax, canola, lentil and field pea), alfalfa, hay and three “other” categories for pulses, oilseeds crops and annual crops that are new or limited in Alberta, Saskatchewan and Manitoba. Each cropping activity can used by one of three tillage practices—CT, MT and ZT—and can be grown in rotation after summerfallow, cereal, pulses, oilseeds, alfalfa, hay or green manure. Crop activities impacts on GHG emissions are modeled to reflect basic agronomic restrictions of crop production in AB, SK and MB and consider the impacts of annual and previous year’s crop rotations and land management practices.

Data was obtained from a number of sources. Seeded area and crop yield data by crop district from 1985 (base year) to 2016 came from various Canadian Socio-Economic Information Management System (CANSIM) Statistics Canada data series. Data on the area under CT, MT, ZT and summerfallow practices are from Statistics Canada’s Census of Agriculture for various years [13] and from industry surveys conducted between census years [18, 19]. The yearly data on inputs used (i.e., fertilizer and fuel) are from Statistics Canada (various years). More information about the source of data is presented below.

Each cropping activity employs a vector of coefficients that represent the environmental measures per hectare for each type of crops and management practices and in each soil-climatic zone. These coefficients are obtained from published literature, as discussed together with the method of measure in the following:

1. *Soil carbon stock (SCS)*:
  *S*oils can be either a source of or sink for atmospheric CO_2_, depending on current and previous land use practices. This source or sink behaviour is mainly induced by the photosynthetic process, the incorporation of crop-residue organic matter into the soil (CO_2_ sink or sequestration), and the decomposition of that organic matter by soil organisms (CO_2_ source of emission) [20]. The soil carbon stock (SCS), which reflects the state of balance between carbon lost through the decomposition of soil organic matter (SOM) and carbon puts into the soil through the incorporation of crop residue [21]
  The use of ZT has shown to promote carbon sequestration and increase SCS [22, 23]. ZT is defined as a system of planting crops into untilled soil that leaves at least 30% of previous crop residue on the soil (or at least 1.1 Mg/ha of crop residue), uses specialized seeding equipment to place seed and fertilizer in the soil minimum soil disturbance, controls weeds by using herbicides, and uses crop rotations to help improve land structure, break the life cycles of pests and diseases, and help in controlling weeds [24, 25].
  Field experiments and comprehensive analyses indicate that SCS increases with the reduction or elimination of tillage practices [26]. Previous studies such as Mangalassery, et al. [22], Lal [27], Follett [28] and Paustian et al. [23] indicated that, with conventional tillage as a baseline, SCS/carbon sequestration significantly increases under ZT and that this increase is lower under MT. The widespread adoption of ZT on the Prairies has enabled higher carbon return to soil through more intensified crop rotations, residue retention, and reduced SOM decomposition rates associated with lower summerfallow and the greater use of conservation tillage practices. Campbell, et al. [29] measured the effect of cropping frequency on SCS under different tillage systems in various agronomic and climate settings in the midwestern USA and the Canadian Prairies. The authors found in the Canadian Prairies that the gain in SCS was higher when using ZT and crop-crop rotation (continuous cropping) in humid and subhumid environments, compared to a lower gain when using CT in semiarid environments regardless of the cropping frequency. McConkey, et al. [30, 31] measured the change in SCS on a network of 137 fields across Saskatchewan that were converted from CT to ZT in different soil type zones. The authors found a significant and consistent increase in SCS under ZT especially in the long run. This increase is greater in the subhumid areas (Thin-black, Thick-black soil zones) than in the arid areas (Brown soil zones). Moreover, ZT improves soil water storage capacity, which affects the amount of crop residues produced, increases soil organic matter and biomass, and thus, leading to an increase in SCS [32]. Crop rotations which have replaced summerfallow contribute to greater carbon sequestration, as land under summerfallow lost soil organic carbon due to the intensive use of tillage practices.
  The soil carbon coefficients induced by tillage practices for each crop and climate zone were obtained from several studies in Western Canada. These coefficients range between 0.83 tonne and 0.92 tonne of CO_2_eq ha^-1^ year^-1^ for a crop-crop rotation, between 0.73 and 2.2 tonne of CO_2_eq ha^-1^ year^-1^ for a reduced fallow-rotation, and between 0.18 and 0.83 of tonne of CO_2_eq ha^-1^ year^-1^ for a fallow-crop rotation [29–31] (A detailed description of the coefficients for every soil type is presented in Table S1.2 in S1 Table). The assumption in this study is that tillage causes CO_2_ to be released into the atmosphere, leading to a decline in SCS. To account for this, we used a positive value of the soil carbon coefficients. Correspondingly, when tillage practices are eliminated (i.e., when using ZT), a negative value of the coefficients is used, indicating CO_2_ removal from the atmosphere (carbon sink/sequestration of emission) and thus, an increase in SCS.
  The soil carbon coefficients are adjusted to account for variable residue retention, such that below-average crop yields reduce the amount of soil carbon sink/sequestration while above-average crop yields increase the rate of sequestration. To measure the amount of crop residue, we followed Fan et al. [21] (a description of Fan et al.’s method is provided below), who estimated the amount of crop residue as a function of crop yield and the harvest index. The coefficient of carbon added to the soil from crop residue (for above- and below-ground biomass) is estimated by the International Panel for Climate Change (IPCC) [33] and Maillard et al. [34] to average 0.45, but this rate accounts for biomass removal or burning but does not include factors associated with the impact of long-term changes in temperature and precipitation. Therefore, a more conservative coefficient that is equal to 0.3 is used in this study. Eq (1) is used to estimate the change in SCS.
$${SCS}_{t}=\sum_{i=1}^{n}\sum_{j=1}^{122\;}{[A}_{ijt}\times {CT}_{ij}]\times \left[R_{ijt}{\times CR}_{ij}\right]{\times CO}_{2}MW$$ (1)
  where *SCS*_*t*_ is the change in soil carbon stock in year *t*; *A*_*ijt*_ is the area (hectares) of crop activity *j* in soil zone *i* in year *t*; *CT*_*ij*_ is the soil carbon coefficient (metric tonne C ha^-1^year^-1^); $R_{ijt}=\frac{Y_{ijt}}{{HI}_{ijt}}\times {(1-Y}_{ijt})$ is Fan et al.’s [21] crop residue level estimated using the total biomass produced from harvested yield, where *Y*_*ijt*_ is the amount of crop yield in metric tonne ha^-1^ and *HI*_*ijt*_ = *α*_*ij*_ + *β*_*ij*_ × *Y*_*ijt*_ is the harvest index of crop activity *j* in soil zone *i* in year *t*, where *α*_*ij*_ is the intercept and *β*_*ij*_ is the coefficient that denotes the relationships between harvest index and crop yield [21, 33] (A full description of the intercept and slope to measure the harvest index for the major crops grown is presented in Table S1.3 in S1 Table); *CR*_*ij*_ is the rate of crop residue input carbon into soil, which, as previously indicated, is assumed to be equal to 0.3; and *CO*_2_*MW* = the ratio of molecular weight of CO2 to C (= 44/12, metric tonne CO2 (metric tons C)^-1^).
  The SCS coefficients in this study collectively capture the effects of tillage practices, cropping systems, soil cover, and crop and soil types. For instance, the coefficient of 2.2 tonne of CO_2_eq ha^-1^ year^-1^ (Table S1.2 in S1 Table) indicates that when a crop is produced using ZT, crop-crop rotation, and grown in the Thick-black or Grey soil zone, SCS increases by 2.2 tonne of CO_2_eq ha^-1^ year^-1^. This coefficient is then adjusted to account for residue retention for each crop type using information from Table S1.3 in S1 Table.
  In this study, we followed the pool approach, which assumes that the capacity of soil to store carbon is infinite. Under this approach, SCS increases linearly with carbon puts, reaching a new level of carbon equilibrium without showing any sign of saturating behavior [35, 36]. The question of SCS saturation has resulted in considerable controversy in the literature [34–37]. For instance, while Paustian [35] and Blair et al. [36] found that SCS saturation is infinite, Campbell et al. [37], Chan et al. [38] and Maillard et al. [34] found that for a certain level of carbon put, soil carbon levels tend to reach an equilibrium, limiting the amount and duration of additional SCS storage. They found that by using improved land use practices, a full carbon storage is achieved in 20 to 50 years. The cycle of carbon is complex with several factors that can impact the capacity of soil to store carbon. Inputs such as nitrogen fertilizer and the amount of crop residue, along with factors such as soil temperature and precipitation affect the yearly amount of carbon that can be stored by the soil.
2. *Emissions from fertilizer application*:
  N_2_O emissions are directly related to the quantity of nitrogen (N) fertilizer added to soils (compared to other fertilizers such as phosphorus and potassium, nitrogen is widely used in the Canadian Prairies as it is considered the most important nutrient to improve a plant’s biochemical and physiological functions, proper plant growth and development and improvement in yield quantity and quality). N_2_O is primarily produced as a result of biotic processes, namely nitrification and denitrification, which are impacted by the rate of N fertilizer applied, the type of soil, soil moisture, crop activities, and the placement of nitrogen fertilizer into the soil. Eq (2) is used to measure the annual emission from N fertilizer application.
$${N_{2}O\_N}_{t}=\sum_{i=1}^{n}\sum_{j=1}^{122}A_{ijt}\times N_{ij}\times {CN}_{i}\times N_{2}OMW$$ (2)
  where N_2_O_N_t_ is the emission from the application of N fertilizer in year t, A_ijt_ is the area of crop activity j in soil zone i in year t, N_ij_ is the nitrogen rate-requirements, CN_ij_ is the N_2_O emission coefficient of crop activity j in soil zone i, and N_2_OMW is the ratio of molecular weights of N_2_O to N2O-N = 44/28 (metric tonne N2O (metric tonne N2O-N)-1).
  The coefficients used to estimate N_2_O emission from N application are adopted from Rochette et al. [39], ECCC [1] and the IPCC Tier 1 default emission factor derived by Bouwman [40] for the Prairies region. Rochette et al. [39] identified key soil, climate factors and management practices that affect N_2_O emissions by compiling soil N_2_O flux a long-term soil data in Canada. The authors found that factors such as growing season precipitation, temperature, crop type, and soil pH, texture and organic carbon affected N_2_O emissions. Bouwman [40] analyzed factors affecting the N_2_O emissions, including soil conditions, type of crop and nitrogen fertilizer type and rate, soil type, and crop management. The authors conducted their analysis by using published measurements of N_2_O emissions from N fertilizers. For the Brown and Dark-brown soils, the coefficient is equal to 0.0016 kg N_2_O-N/kg N, and in the Grey and Black soils it is equal to 0.003 kg N_2_O-N/kg N. These coefficients imply that N_2_O emissions from N application in the Canadian Prairies region increase with increased moisture in well-aerated soil types, such as Grey and Black soils. To capture the impact of tillage practices on N_2_O-N emissions, the coefficients are reduced by 20% in the case of ZT [41, 42]. Rochette et al. [41] found that in the Prairies, when ZT is used, N_2_O emissions can be reduced when placing N fertilizer near the zone of active root uptake; the authors confirmed that N_2_O emission from N fertilizer under ZT is 20% lower than under CT.
  The rates of nitrogen fertilizer by soil zone for cereal and oilseed are estimated using data obtained from [43, 44]. These rates range between 19.5 and 136.7 kg N ha^-1^. For lentils, field peas and other pulse crops, which receive nitrogen and phosphorus together, a rate of 2.5 kg N ha^-1^ is assumed to be applied to all seeded areas in all soil-climate zones.
3. *Emissions from crop residue retention*:
  Eq (3) is used to account for the nitrification and denitrification of the N released during the decomposition of crop residues and the resulting impact on the release of N_2_O emission into the atmosphere.
$${N_{2}O\_R}_{t}=\sum_{i=1}^{n}\sum_{j=1}^{122}R_{ijt}\times \left({NA}_{j}{\times RA}_{j}+{NB}_{j}{\times RB}_{j}\right)\times NR\times N_{2}OMW$$ (3)
  where *N*_2_*O*_*R*_*t*_ is the emission from crop residues above and below ground in year *t*, *R*_*ijt*_ is the crop residue level (see Eq (1) for definition); *NA*_*j*_ and *RA*_*j*_ are N content and the ratio of above-ground residues to harvest yield for crop *j*, respectively; *NB*_*j*_ and *RB*_*j*_ are N content and the ratio of below-ground residues to harvest yield for crop *j*, respectively. *NR* is the emission coefficient used for all sources of N_2_O emissions from agricultural soils, obtained from IPCC (2006) and is equal to 0.0125 kg N_2_O-N/kg N. Nitrogen content of above-ground and below-ground residues, and the ratios of below- and above-ground residues to harvested yield, are obtained from [33] (A full description of these rates for the major crops is presented in Table S1.4 in S1 Table).
4. *Emissions from summerfallow practice*:
  Although N fertilizer is not applied during the summerfallow period, several factors may stimulate N_2_O production from fallow, including higher soil water content, temperature, soil carbon and nitrogen. Following Rochette et al. [41], we estimated the N_2_O emissions from fallow as the sum of the N_2_O emissions from the previous year’s N application and crop residue multiplied by the fraction of land that is under summerfallow for each crop soil zone. Eq (4) is used to estimate the emission from summerfallow:
$${N_{2}O\_S}_{t}=\sum_{i}^{n}(N_{2}O\_N_{it}+N_{2}O\_R_{it})\times FS_{it}$$ (4)
  where *N*_2_*O*_*S*_*t*_ is *N*_2_*O* emissions due to the summerfallow practice in year *t*. *N*_2_*O*_*N*_*t*_ and *N*_2_*O*_*R*_*t*_ are the *N*_2_*O* emissions due to the summerfallow practice and fertilizer nitrogen application in year *t*, measured in Eqs (2) and (3), respectively. *FS*_*it*_ is the fraction of cropland that is under summerfallow in crop soil zone *i* in year *t*.
5. *Emissions from fuel used for crop production and transportation*:
  Automotive fuel is used during seeding, crop protection and harvest operations. The rates of fuel consumption for different types of powered equipment (gigajoules (GJ) ha^-1^), is obtained from [45]. Gill et al. [45] developed a method to estimate and compare non-renewable energy inputs, energy outputs and energy use efficiency generated from the production of crops in the Canadian Prairies (the following factors were included in Gill et al.’s [45] analysis: nitrogen use, ZT, summerfallow, crop rotations, fuel of farm machinery, and crop diversification and extension); the coefficients were generated in terms of energy value of fuel used (diesel and gasoline) in the cropping activities in the Prairies. The emission coefficient is assumed to be equal to 74.06 g/MJ, a value obtained from [46], which represents the rate of CO_2_ emitted from powered agricultural equipment.
  The model also includes energy used for transportation of crop inputs and outputs; the fuel coefficients used in the model were developed by the Agriculture Canada Research Centre using crop inputs and outputs data along with energy consumption rates for powered equipment obtained from [16, 45] Consumption rates were developed assuming a 25 km round trip for crop inputs and grain sales. To account for the increase/decrease in crop production and hauling distance for each year, the energy consumption rates were adjusted using data obtained from [47–49]. Eq (5) is used to measure emissions from fuel used for crop production and transportation.
$${CO}_{2}\_F_{t}=\sum_{i=1}^{n}\sum_{j=1\;}^{122}[(A_{ijt}\times {FC}_{1})+\;(A_{ijt}\;\times \;{FC}_{2}\;+\;Y_{ijt}\;\times \;{FC}_{3})]\;$$ (5)
  where *CO*_2__*F*_*t*_ is the *CO*_2_ flux to the atmosphere caused by energy use and fossil fuel consumption in year *t*, *A*_*ijt*_ and *Y*_*ijt*_ are the area (hectares) and crop production of crop activity *j* in crop zone *i* in year *t*, respectively. *FC*_1_ is the energy coefficient for powered equipment used on farm, *FC*_2_ is the energy coefficient that reflects the distance to transport outputs and inputs, and *FC*_3_ are energy coefficients that reflect the size of crop production.
  All data and coefficients used in quantifying GHGs carry an intrinsic level of uncertainty. Uncertainty in measuring GHGs increases when farm-tested data are not used. This arises from the fact that each farm has a unique combination of management practices, crop types, soil and climate conditions. Consistent detailed farm-level data over the study period is unavailable. Therefore, to reduce this uncertainty, data at the soil-climate zones for each of the 122 crop activities is used in this study.
  Another source of uncertainty relates to the GHG emission/sink coefficients that are used in the model. Ideally, geographic-specific field estimated coefficients are required to quantify GHGs. In this study, to reduce uncertainty, efforts have been made to choose the model coefficients that are based on specific soil properties and crop systems. However, fuel coefficients are not available at the soil type and for each cropping activity; therefore, these coefficients were applied to a broader geographic area and to the wide range of cropping systems. Moreover, when measuring crop residue retention emissions, we used coefficients that are not based on Canadian field measurements, but are instead obtained from international research (i.e., IPCC). These add uncertainty to the estimates due to the unknown nature of their applicability to the soil-climate zones in the Canadian Prairies.
  As more studies are conducted in the future and more coefficient estimates become available, the model may be revised to use soil-climate zone or farm field level coefficients that are specific to the Canadian Prairies. Moreover, as more GHG coefficients will become available in the future, a range of plausible coefficient estimates might be considered, and computer simulating techniques (i.e., Monte Carlo simulation) could be conducted to deal with the uncertainty about the coefficients values and to produce a distribution of possible GHG measures.

## Results and discussion

**Fig 3:**
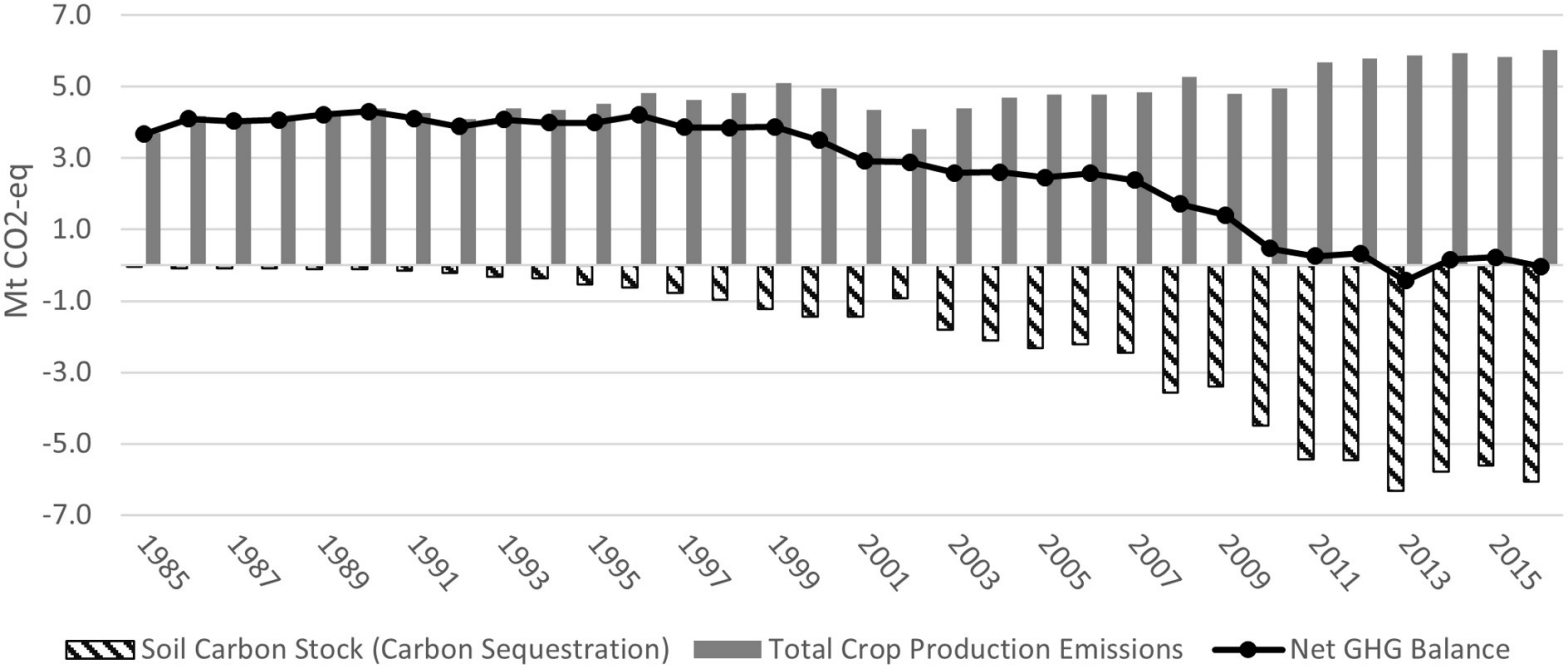
Alberta GHGs estimates in the crop sector (Mt CO_2_-eq) (1985–2016).

**Fig 4:**
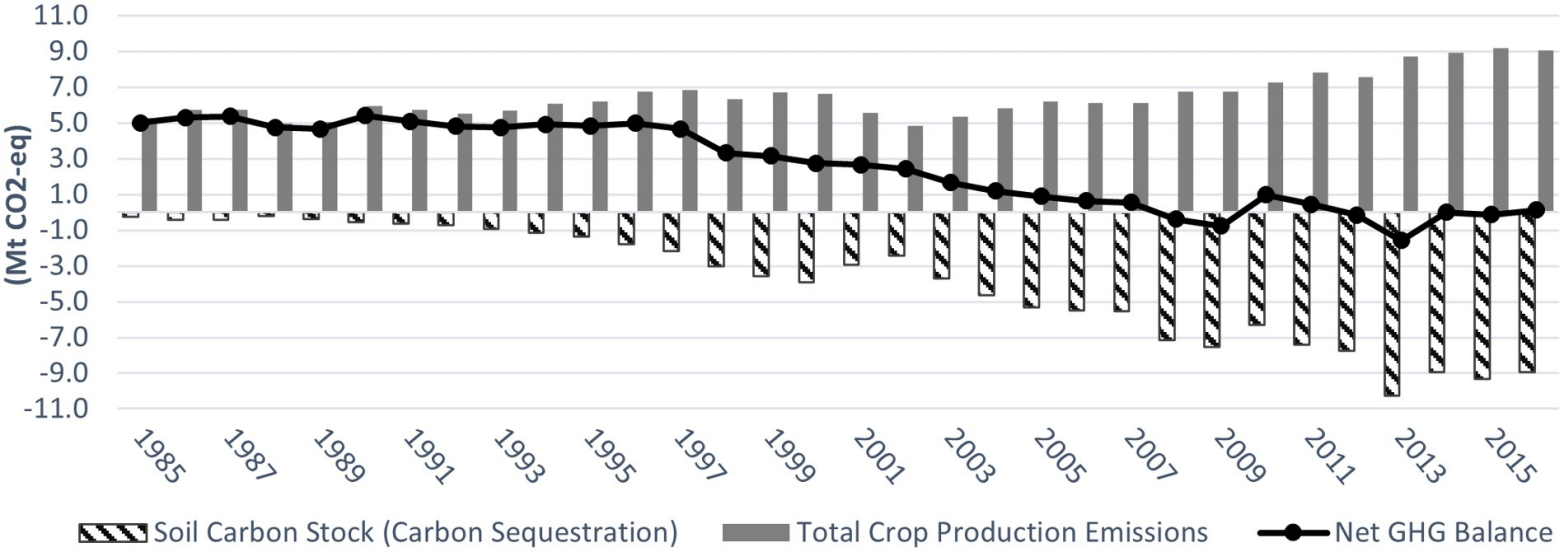
Saskatchewan GHGs estimates in the crop sector (Mt CO2-eq) (1985–2016).

**Fig 5:**
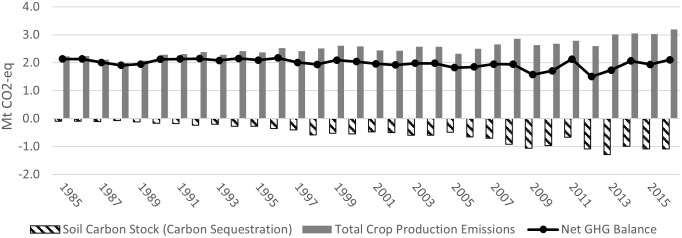
Manitoba GHGs estimates in the crop sector (Mt CO2-eq) (1985–2016).

**Fig 6:**
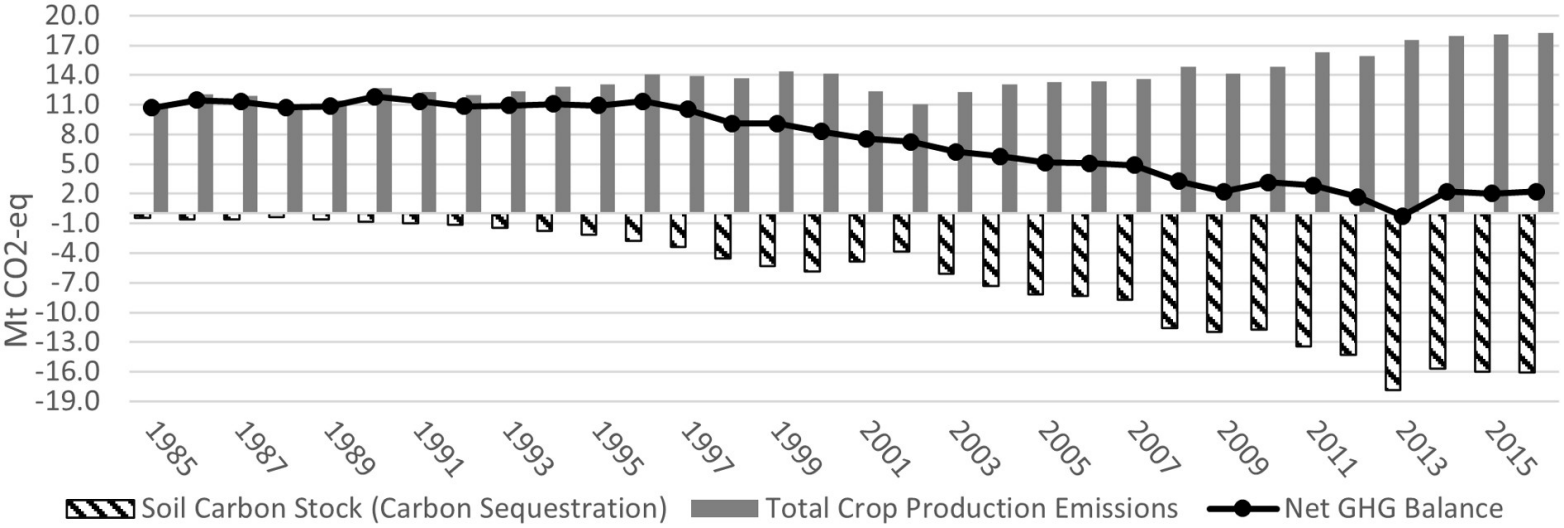
Canadian Prairies GHGs estimates in the crop sector (Mt CO2-eq) (1985–2016).

Figs 3–6 show the GHGs estimates in Alberta, Saskatchewan, Manitoba, and in the Canadian Prairies (aggregate level), from 1985 (base year) to 2016, respectively. Table 1 summarizes the results and compares the 2016 estimates to those of 2005 and 1985.

**Table 1:** GHGs estimates in the Canadian Prairies provinces in the crop sector (Mt CO2-eq).

GHGs Sources & Sink	Alberta			Saskatchewan			Manitoba			Prairies		
	1985	2005	2016	1985	2005	2016	1985	2005	2016	1985	2005	2016
**Soil carbon stock (SCS)**	(0.05)	(2.32)	(6.06)	(0.26)	(5.34)	(8.94)	(0.10)	(0.49)	(1.09)	(0.41)	(8.15)	(16.1)
**Fertilizer**	0.99	1.10	1.59	0.76	1.21	2.25	0.77	0.85	1.32	2.52	3.16	5.16
**Residue**	1.07	1.67	1.99	1.51	2.36	2.73	0.74	0.57	0.89	3.32	4.60	5.61
**Fallow**	0.40	0.23	0.07	0.88	0.58	0.19	0.10	0.22	0.02	1.38	1.03	0.28
**Total Fuel**	1.26	1.76	2.37	1.98	2.07	3.90	0.63	0.72	0.97	4.87	4.54	7.25
** • Fuel on farm**	1.06	0.76	0.76	1.84	1.42	1.26	0.50	0.37	0.38	3.4	2.55	2.4
** • Fuel for transp**.	0.20	1.00	1.61	0.15	0.64	2.65	0.12	0.35	0.59	0.47	1.99	4.85
**Net GHG**	3.67	2.38	(0.04)	4.87	0.88	0.13	2.14	1.87	2.11	10.81	5.13	2.20

### Soil Carbon Stock (SCS)

Figs 3–5 (dashed bars) show that from 1985 to 1989, soil carbon stock (SCS) was increasing (absolute value), albeit at a slowing rate, ranging between -0.048 and -0.098 Mt CO_2_eq in Alberta, between -0.26 and -0.37 Mt CO_2_eq in Saskatchewan and between -0.097 and -0.117 Mt CO_2_eq in Manitoba, respectively (the negative sign indicates net carbon sink/sequestration). At the Prairie level, Fig 6 shows that, during the same period, net SCS averaged between -0.41 and -0.58 Mt CO_2_eq. The low historical level of SCS was mainly due to the lengthy use of tillage operations and the resulting loss in SOM. As soil is tilled, top layers are turned over, air mixes in, and soil microbial activity increases over baseline levels. As a result, SOM is broken down more rapidly, and carbon from the soil is disbursed into the atmosphere as CO_2_ [50].

SCS has increased in the Prairie provinces during most of the years under study (Fig 6). In Alberta SCS increased from -0.103 Mt CO_2_eq in 1990 to -2.325 Mt in 2005 and to -6.056 Mt CO_2_eq in 2016 (Fig 3). Over the period of 1985 to 2016, Albert’s total SCS was -66.462 Mt CO_2_eq. The total value of SCS for Alberta (in 2018 dollars, deflated using the consumer price index) for the 31year period was C$281.2 million if a tonne of CO_2_eq was worth C$5, C$562.3 million at C$10/tonne and C$843.5 million at C$15/tonne [51]. At a 5% discount rate, the net present value of SCS over the 1985–2016 period is C$204.4 million, C$408.8 million and C$613.3 million at the three reference prices (similar results were reported by [5]) (Alberta’s yearly SCS quantities and values are in Table S2.1 in S2 Table).

In Saskatchewan net SCS went from -0.57 Mt CO_2_eq in 1990 to -5.3Mt in 2005 and to -8.9 Mt CO_2_eq in 2016 (Fig 4). For the entire 31-year period, total SCS was -125 Mt CO_2_eq (Fig 4). Using the same reference prices as in Alberta, the total value of SCS for the 31years totaled C$0.514 billion (at C$5/tonne), C$1.028 billion (at C$10/tonne) and C$1.542 billion at C$15/tonne). Using a 5% discount rate, the net present values of SCS would be C$0.35 billion, C$0.71 billion and C$1.1 billion, at the three reference prices (see Table S2.2 in S2 Table).

The increase in SCS in Alberta and Saskatchewan can be attributed to the widespread adoption of ZT, which enabled the recovery of historical losses of soil carbon caused by CT. The use of ZT practice increased from around 3.7% of Alberta’s total cropland in 1985 to 29.7% in 2005 and to 67.5% in 2016. In Saskatchewan, the use of ZT increased from 4.7% of total cropped land in 1985 to 51% in 2005 and to 74.5% in 2016 [13, 18, 19]. The adoption of ZT has not only improved SCS, but also enhanced soil quality (increased soil moisture and organic matter and decreased soil and tillage erosion and salinity) and productivity [15, 52–56].

In Manitoba (Fig 5, dashed bars) net SCS ranging from -0.164 Mt CO_2_eq in 1990 to -0.49 Mt in 2005 and to -1.08 Mt CO_2_eq in 2016, albeit with reversals in 1999, 2005, 2011 and 2014 when flooding and wet springs required increase use of tillage and summerfallow to dry the fields (similar results were reported by [5]). For the 31-year period, net SCS totaled -17.4 Mt CO_2_eq, generating a gross value of C$69.6 million, C$139.3 million or C$209 million, depending on the reference price for carbon (see Table S2.3 in S2 Table).

Relative to Alberta and Saskatchewan, the increase in Manitoba’s SCS was not significant. This is mainly due to the low rate of ZT adoption in Manitoba, which rose from 5% in 1985 to 21% in 2005, before settling at 20% in 2016 [13, 18, 19]. A number of factors limit adoption of ZT in Manitoba. First, since 1999, western Manitoba has experienced abnormally high annual precipitation, in combination with severe flood events in 2011 and 2014, that led farmer to undertake more tillage and to incorporate crop residue into the soil to dry land out in order to plant crops. Second, the soil near Carberry, Manitoba, is very fine sandy loam and has recently been cultivated in a potato/cereal rotation. The production of potatoes requires intensive multiple tillage operations in the season and extra tillage after harvest to level the land so cereals can be seeded in the subsequent season. Third, in eastern Manitoba, soils are high in clay and frequently flooded, which forces farmers to increase tillage practice, allowing the soils to dry and warm up more quickly for spring seeding. Fourth, Manitoba farmers have replaced durum, lentils, and field peas with more long-season, heat-loving crops such as soybeans and corn, all which require intensive tillage in the early spring to warm up the soil.

At the soil zone level, the largest increases in SCS were recorded in dark-brown soils. In Alberta, Dark-brown soils, accounting for around 28.5% of the total SCS, compared with Gray (20.5%), Thin-black (20.3%), Thick-black (19%) and Brown soil types (11.6%) (Table S2.1 in S2 Table). In Saskatchewan, Dark-brown soils accounted for 31.1% of the total SCS, followed by the Brown (21%), Thick-black (19.6%), Gray (14.6%) and Thin-black soil types (14%) (Table S2.2 in S2 Table). In Manitoba, increase in SCS was mostly in Thin-black (61.4%) and Thick-black (34.6%) zones (Table S2.3 in S2 Table).

### Total GHG emissions

Figs 3–5 (solid bars) show the estimates of total gross GHG emissions from cropping activities in Alberta, Saskatchewan, and Manitoba between 1985 and 2016, respectively. Total gross emissions in Alberta increased from 3.72 Mt CO_2_eq in 1985 to 4.78 in 2005 and 6.0 Mt CO_2_eq in 2016, in Saskatchewan from 5.13 Mt CO_2_eq in 1985 to 6.22 in 2005 and 9.07 Mt CO_2_eq in 2016, and in Manitoba from 2.23 Mt CO_2_eq in 1985 to 2.31 in 2005 and 3.19 Mt CO_2_eq in 2016. At the Prairie level, Fig 6 shows that total GHG emissions increased from 11.1 Mt CO_2_eq in 1985 to 13.3 in 2005 and 18.3 Mt CO_2_eq in 2016. Total gross GHG emissions includes the emission generated from fertilizer application, residue retention, summerfallow and fuel (Awada and Nagy [5] reported similar emissions results in Alberta and Manitoba):

1. ***Fertilizer emissions***: Alberta’s emissions from fertilizer application increased by 60.4% between 1985 and 2016, Saskatchewan’s nearly increased by threefold between 1985 and 2016 and Manitoba’s more than 71.1% (Table 1). Total Prairie emissions from fertilizer application increased from 2.52 Mt CO_2_eq in 1985 to 3.16 Mt in 2005 and 5.16 Mt CO_2_eq in 2016, as increased crop rotation and reduced summerfallow required greater use of fertilizer. Over the 31 year period, Alberta’s nitrogen use increased by 91%, while crop production increased by 117%. Over the same period, Saskatchewan fertilizer use increased 98% while crop production increased 61% and in Manitoba, fertilizer use increased 77% but crop production increased by only 26% [11, 44]. The lower inputs and outputs in Manitoba reflect the traditionally low levels of summerfallow and relatively more productive soils.
  At the soil zone level, fertilizer emissions in Alberta increased 117% in the Brown soil type 79.7% in Dark-brown soils, 56% in Gray, 50.6% in Thin-black and 48.4% in Thick-black types. In Saskatchewan, fertilizer emissions rose more than 200% in the Brown and Dark-brown soils between 1985 and 2016, followed by Thin-black (197%), Gray (181%), and Thick-black soil types (174%). Manitoba’s fertilizer emissions increased less, rising 93.1% in Dark-brown soils, 83.2% in Thin-black, 68.6% in Gray and 63% in Thick-black soil types (see Tables S2.4 –S2.6 in S2 Table).
  Nitrogen has low nutrient use efficiency, causing high N_2_O fluxes that have led to major environmental impacts. Over the past 150 years, increasing N_2_O emissions have contributed to stratospheric ozone depletion at 2 percent per decade. While typically only 0.5% to 3% of the nitrogen applied is converted to N_2_O emission, this represents a large percentage of the total GHG emissions in the crops sector, since N_2_O has 310 times the global warming potential of CO_2_ (Other forms of emissions from fertilizer nitrogen may also be released into the environment, for instance, NH3, and NO3), referred to as indirect N_2_O emissions, that can be transferred into N_2_O in downwind or downstream ecosystems) [57, 58]. This is not sustainable as large-scale, long-term fertilizer use significantly alters soil nutrient balance and increases both soil acidification and fertility deficiency [59, 60]. A well-developed nitrogen management system—involving variable application rates, optimal timing, placement, and formulation, diversified crop rotations, active MT or ZT, and intensive management of soil pH, pests and disease—is critical for GHG emissions mitigation and for the health and resilience of agricultural land.
2. ***Residue emissions***: Over the period of 1985 to 2016, N_2_O emissions from decomposition of retained residue increased in each province—Alberta (86%), Saskatchewan (81%) and Manitoba (20%). At the Prairies level, these emissions increased from 3.32 Mt CO_2_eq in 1985 to 4.60 in 2005 and 5.61 Mt CO_2_eq in 2016. At the soil zone level, the largest increases were in the Brown soil types in Alberta and Saskatchewan and in Black soils in Manitoba (Tables S2.7-S2.9 in S2 Table). Increased residue emissions arose due to the replacement of summerfallow by continuous cropping under ZT while increased the area covered by crop residue. While the same amount of N_2_O emission is released from the decomposition of retained residue regardless of the cultivation practice, conventional tillage requires multiple passes of machinery which leads to higher CO_2_ emissions for fuel [26].
3. ***Summerfallow emissions***: Between 1985 and 2016, N_2_O emissions from fallow decreased in all provinces by about 80% (Table 1). At the Prairie level, emissions decreased from 1.38 Mt CO_2_eq in 1985 to 1.03 in 2005 and to 0.28 Mt CO_2_eq in 2016. Decreases happened on all soil types, ranging from 75% to 90% (Tables S2.10—S2.12 in S2 Table). There was a significant drop in the area under summerfallow throughout the region. In Alberta, around two million hectares were under summerfallow in 1985; this has continuously dropped to reach 240,000 ha in 2016. In Saskatchewan, 5.9 Mha was fallowed in 1985, but only 2.5 Mha in 2005 and under 600,000 ha in 2016. In Manitoba, summerfallow decreased 50% by 2005 and 90% by 2016 from the base of 0.4 Mha. While the declines were steady in Alberta and Saskatchewan, summerfallow increased in Manitoba in the main flood years (1999, 2011 and 2014) [4]. The combination of new crop varieties (especially canola and pulses), new seed inoculants, land rollers, flexible harvest headers and better agronomic knowledge all combined to improve the economic returns to rotational crop production.

### Fuel emissions

Over the 1985 to 2016 period, C_2_O emissions from all fuels used on farm and for crop related transportation increased in all provinces—Alberta (87%), Saskatchewan (97%) and Manitoba (54%)–and the Prairies level, where emissions rose from 4.87 Mt CO_2_eq in 1985 to 4.54 Mt CO2eq in 2005 and to 7.25 Mt CO_2_eq in 2016 (Table 1) (see Tables S2.13 –S2.15 in S2 Table). This overall impact is made up of two contrasting changes. Emissions from fuel used specifically for crop production decreased due to lower tillage and summerfallow, dropping in all provinces by about 28% (Table 1). At the soil zone level, the decrease in these emissions ranged between 21% and 41% (Tables S2.16 –S2.18 in S2 Table). In contrast, emissions from fuel consumed transporting crop inputs and outputs increased significantly between 1985 and 2016, in Alberta by sixfold, in Saskatchewan seventeenfold, and in Manitoba fivefold (Table 1) (annual estimates are in Tables S2.19 –S2.21 in S2 Table). This increase was due to growth in farm size that increased distances between fields and the home quarter, centralized on-farm storage, a significant consolidation in the number of commercial grain delivery points (Alberta’s delivery points decreased by 82%, Saskatchewan’s by 78% and Manitoba’s by 66%), consolidation of input suppliers in line with the reduced number of delivery points, and decrease in the number of grain elevators by 87% in Alberta, 84% in Saskatchewan, and 73% in Manitoba over the period of 1985 to 2016.

#### Net GHG balance and value

**Fig 7:**
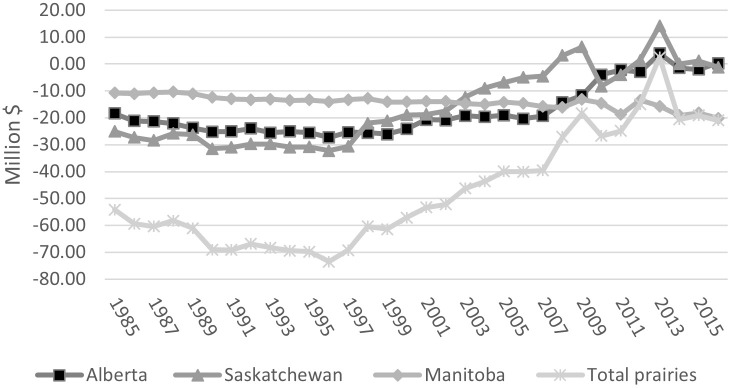
Net GHG value in the Canadian Prairies crop sector, 1985–2016 (price of CO_2_eq/ton = C$10, $2018).

The net GHG balance in each province has improved (net GHG balance = total emission -|SCS|). In Alberta the net balance decreased most years, dropping from the high of 3.7 Mt CO_2_eq in 1985 to 2.5 by 2005 and -0.035 Mt CO_2_eq in 2016 (the negative sign indicates net sink of GHG) (Fig 3, black line). The cropping sector in Alberta was a net sink in two of the last four years estimated –2013 and 2016 –and over the four years in aggregate sequestered carbon (Fig 3). As a result, the value of net GHG emissions (priced at C$10/tonne CO_2_eq) inverted, from a (C$18) million debit in 1985 to a credit equal to C$0.3 million in 2016 (Fig 7) (annual estimates are in Table S2.22 in S2 Table).

Saskatchewan net GHG emissions dropped from 4.87 Mt CO_2_eq in 1985 to 0.88 in 2005 and below zero in 2012–2015. Most importantly, the cropping sector has a cumulative net sink for the past decade (Fig 4, black line). At a carbon price of C$10/tonne CO_2_eq, the value of net GHG decreased from (C$25 million) in 1985 to (C$6.8 million) in 2005, and from 2006 to 2016 a cumulative positive value of C$3.6 million (Table S2.23 in S2 Table).

In Manitoba annual net GHG emissions have stayed at about the same level over most of the 1985–2016 period, with a net balance of 2.1 Mt CO_2_eq in both 1985 and 2016, with the exception of 2009, 2010, 2012 and 2013, when the net GHG balance reached, on average, 1.6 Mt CO_2_eq (Fig 5, black line). Priced at C$10/tonne CO_2_eq, the debit value of net GHG emissions increased from (C$11 million) in 1985 to (C$20 million) in 2016 (Fig 7) (Table S2.24 in S2 Table).

For the Prairie as a whole, net GHG emissions decreased from 10.81Mt CO_2_eq in 1985 to 5.13 Mt CO_2_eq in 2005 and to 2.2 Mt CO_2_eq in 2016. At C$10/tonne CO_2_eq, the value of net GHG decreased from (C$54 million) in 1985 to (C$40 million) in 2005 and to (C$21 million) in 2016 (parentheses indicate a debit balance).

Globally and nationally, a few studies have quantified separate contributions of the crop sector and the livestock sector to total agriculture GHG emissions. For instance, Martin and Hoppe [12] reported a decline of 10% between 1981 and 2011 in Canada’s net agricultural (combined crop and livestock sectors) GHG emissions. They attributed this decline mainly to the adoption of ZT and replacement of summerfallow with crop rotations in the Canadian Prairies, which enabled the crop sector to sequester carbon in soil. They estimated that soil carbon in Canada went from being a source of emissions (1.1 Mt CO_2_eq) in 1981 to being a large carbon sink (-11.9 Mt CO_2_eq) in 2011 [12].

Globally, data from FAOSTAT [61], computed following Tier 1 IPCC [33] guidelines for national GHG inventories, estimated that global cropland net emissions were 18.88 Gt (gigaton) CO_2_eq year^-1^ over 1990–2019, including methane (CH_4_) emissions from rice, CO_2_ emissions from peatland cultivation, and N_2_O emissions from fertilizers applications. The production of cereals, notably paddy rice, represents the largest source of global cropland emissions, accounted for more than 47% of global arable land use emissions in 2017 [62]. Asia, especially Indonesia, contributed the most to global emissions from cropland (57%), followed by Europe (20%), the Americas (10%), Africa (8%) and Oceania (5%). During the same period, all of Canada’s croplands were estimated to have emitted 3 Gt CO_2_eq year^-1^, making Canada the 8^th^ largest cropland emitting country [61]. In the Canadian Prairies, the main driver of continued emissions in the crop sector is the rising application of nitrogen fertilizer, which increased 150% between 1981 and 2011 [12].

Emissions caused by nitrogen use in agriculture have become a global problem. The excessive and inefficient use of nitrogen fertilizer has increased N_2_O emissions by more than 80% between 1980 and 2018 [63]. Nitrogen fertilizers are currently only 25% to 50% efficient. The development and adoption of technologies and agronomic practices that improve fertilizer efficiency and reduce its environmental consequences are needed. The use of controlled-slow-release N fertilizers and the application of the 4R nutrient management principles (right source, right rate, right timing, and right placement) are considered the best current techniques to increase nutrient use efficiency and reduce environmental consequences. Other potential new technologies include nano-enabled growth enhancers, nanofertilizer, and nano-enabled seed coatings. Laboratory studies of nanotechnologies indicate tremendous promise to improve NUE and to make the crop sector more sustainable, efficient and resilient. However, efficient delivery at field scale is considered a barrier for the implementation of nano-enabled technologies in agriculture [64].

Globally, GHG emissions from energy use in agriculture have increased by more than 100% between 1980 and 2018 [65]. The rate of emissions from energy consumption by agricultural operations has increased more rapidly in developing countries than that of developed countries. This is due to the greater reliance on fossils fuels, notably coal, oil, and natural gas in developing countries and the increased shift towards low-emissions and renewable energy sources (e.g., hydro, solar, wind, and bioenergy) in developed countries [66, 67]. Several studies have found that the adoption of low-emissions energy technologies, renewable energy, and energy-efficient management practices in agriculture has the potential to reduce GHG emissions caused by energy consumption in developing countries [68, 69]. Ali et al. [68] and Ali and Abbas [69] found that the use of electricity and gas-based equipment instead of petroleum-based equipment by the agricultural sector has reduced CO_2_ emissions in Faisalabad, Pakistan, between 1971 and 2010. The results of the mitigation potential analysis reported in Ali et al. [70] provides evidence that the adoption of low-emission energy technologies can significantly reduce GHG emissions in developing countries to help meet the 2050 goals. Ramírez-Contreras [71] found that renewable energy in the form of bioenergy has the potential to reduce fossil fuel dependence in the Orinoquia region of Colombia by 2030, offering the opportunity to slow down the rate of GHG emissions in this region. Moreover, using proper technology platforms (e.g., geospatial technology, and precision agriculture) with perennial bioenergy crops can not only increase feedstock supply for renewable biofuel production but also improve soil health, sequester carbon, and provide habitat for pollinators and other wildlife species [66, 72, 73]. From an economic perspective, integrating bioenergy crops in the production system creates new markets for farmers and generates additional farm revenue and new employment opportunities [66].

Today, fast-evolving sensor-based digital data, internet-of-things technologies and big data integration, analysis, and predictive modelling in smart farming or digital agriculture are claiming to improve farmers’ decision-making and the efficiency of input use, and promising to provide innovative solutions for balancing among multiple interlinked agricultural goals, including: mitigating agricultural GHG emissions; preventing the depletion of natural resources; strengthening agricultural resilience to climate change; and addressing the relationship between agricultural productivity, farm incomes and food insecurity [74].

## Conclusion and remarks

The crop sector in the Canadian Prairies has significantly reduced GHG emissions since 1985. This study measures the progress toward reducing emissions by measuring the long-run contribution of different crop production practices and inputs’ usage to GHGs on the Prairies for the period 1985–2019. The GHG estimates are conducted at the disaggregated level for each of the soil climate zones and provinces (Alberta, Saskatchewan and Manitoba), and at the aggregated level for the Canadian Prairies region. The estimates of GHGs were then converted to real dollars to measure the social value of these changes.

The reduction and in places elimination of tillage and replacement of summerfallow with crop rotations enabled the crop sector to increase soil carbon stock (SCS) in the Canadian Prairies. Consequently, the net balance of GHGs decreased by 80% between 1985 and 2016 and by 53% between 2005 and 2016. At the provincial level, the crop sector was a net GHG sink between 2013 and 2016 in Alberta and for the decade between 2006 and 2016 in Saskatchewan. In Manitoba, net GHG emissions were largely unchanged between 1985 and 2016, albeit with some volatility. Compared to Alberta and Saskatchewan, Manitoba faced different agronomic options that limited their adoption of conservation tillage practices. At a C$10/tonne CO_2_eq price, the social cost of net GHG on the Canadian Prairies decreased from (C$54 million) in 1985 to (C$21 million) in 2016.

The results of this study provide strong evidence that sustainable agricultural practices can and do reduce GHG emissions. The widespread adoption of sustainable practices (i.e., ZT) in Alberta and Saskatchewan enhanced soil carbon sequestration and changed the crop sector from being a GHG emitter to being a large carbon sink. This change has significantly exceeded Canada’s commitments to the 21st Conference of the Parties (COP21) in Paris, which targeted first to cut net GHG emissions 30% by 2030 and now targets a 50% reduction below 2005 levels.

While the improvements are laudable, more can and probably should be done. This study identifies three areas where emissions might be further reduced: (1) from tillage practices in Manitoba; (2) from nitrogen fertilizer application; and (3) from fuel used for tillage and to transport inputs and outputs. These priority areas need further research to identify and develop sustainable technologies and practices to simultaneously mitigate emissions, increase resilience and ensure low-carbon agricultural productivity growth.

From a policy making standpoint, effective climate change policies require measured evidence to drive choices that support both more effective climate policy and reduce unintended consequences. As Peter Drucker famously wrote, “what gets measured gets managed” [75]. Quantifying the contribution of farming practices to GHGs is key to identifying, managing and mitigating the environmental impacts in the agricultural sector. As this paper has demonstrated, the agricultural system is complex. Detailed modelling is required to tease out the trade-offs between the multiple objectives of farm policies. Clearly, the farm sector has significant potential to mitigating the contribution of its activities to GHG emissions. But industry and governments also responsible for developing strategies to build resilience in agriculture and food systems, increase agricultural productivity to sustain farm profitability, to secure our global food supply and, ultimately, to support sustainable low-carbon economic growth.

## Supporting information

S1 Table Data and coefficients used in the Prairie Crop Energy Model (PCEM). Percentage of arable land in soil-climate zone at the crop district level in Alberta, Saskatchewan, and Manitoba. Soil carbon coefficients by soil-climate zone used in the PCEM. Intercept and slope used in the PCEM to measure the harvest index for the major crops in the Canadian Prairies. Nitrogen content and ratio of aboveground and belowground residues of the major crops grown on the Prairies.
(DOCX)

S2 Table Annual estimates of GHG emissions and sinks at the soil-climate zone and provincial levels. Annual soil carbon stock (SCS) quantities and values. Annual emissions from fertilizer N application, residue retention, summerfallow, and fuel (crop production and transportation). Annual net GHG balance and values in Alberta, Saskatchewan, and Manitoba.
(DOCX)
