# Supplementary material for: Contribution of land use practices to GHGs in the Canadian Prairies crop sector
Source: PLoS One. 2021 Dec 17;16(12):e0260946. doi: 10.1371/journal.pone.0260946 (PMC8682883; doi:10.1371/journal.pone.0260946)
Supplement: S1 Table — Percentage of arable land in soil-climate zone at the crop district level in Alberta, Saskatchewan, and Manitoba. Soil carbon coefficients by soil-climate zone used in the PCEM. Intercept and slope used in the PCEM to measure the harvest index for the major crops in the Canadian Prairies. Nitrogen content and ratio of aboveground and belowground residues of the major crops grown on the Prairies. (DOCX) [file pone.0260946.s001.docx]

**S1 Table: Data and Coefficients used in the Prairie Crop Energy Model (PCEM)**

**Table S1.1. Percentage of arable land in soil-climate zone at the crop District level**

| **Alberta Crop Districts** | Brown | Dark Brown | Thin Black | Thick Black | Gray |
| --- | --- | --- | --- | --- | --- |
| 1 | 100% | 0% | 0% | 0% | 0% |
| 2 | 20% | 80% | 0% | 0% | 0% |
| 3 | 0% | 10% | 70% | 20% | 0% |
| 4 | 0% | 44% | 46% | 10% | 0% |
| 5 | 0% | 0% | 10% | 80% | 10% |
| 6 | 0% | 0% | 0% | 20% | 80% |
| 7 | 0% | 0% | 0% | 0% | 100% |

| **Saskatchewan crop district** | Brown | Dark Brown | Thin Black | Thick Black | Gray |
| --- | --- | --- | --- | --- | --- |
| 1 | 0% | 33% | 67% | 0% | 0% |
| 2 | 3% | 86% | 11% | 0% | 0% |
| 3 | 84% | 16% | 0% | 0% | 0% |
| 4 | 100% | 0% | 0% | 0% | 0% |
| 5 | 0% | 1% | 30% | 56% | 14% |
| 6 | 0% | 84% | 16% | 0% | 0% |
| 7 | 43% | 54% | 3% | 0% | 0% |
| 8 | 0% | 9% | 0% | 38% | 53% |
| 9 | 0% | 1% | 0% | 52% | 47% |

| **Manitoba Crop Districts** | Brown | Dark Brown | Thin Black | Thick Black | Gray |
| --- | --- | --- | --- | --- | --- |
| 1 | 0% | 0% | 90% | 10% | 0% |
| 2 | 0% | 5% | 35% | 40% | 20% |
| 3 | 0% | 0% | 0% | 100% | 0% |
| 4 | 0% | 0% | 10% | 90% | 0% |
| 5 | 0% | 0% | 0% | 100% | 0% |
| 6 | 0% | 0% | 0% | 70% | 30% |
| Source: Authors’ calculation | | | | | |

#### Table S1. 2. Soil carbon coefficients by soil type (Mg CO_2_-eq ha^-1^)

|  | Elimination of Fallow | Direct seeding Fallow greater > 25% | Direct seeding Continuous Crop |
| --- | --- | --- | --- |
| Brown | 0.73 | 0.83 | 0.83 |
| Dark brown | 1.10 | 0.83 | 0.83 |
| Thin black | 1.83 | 0.18 | 0.92 |
| Thick black | 2.20 | 0.18 | 0.92 |
| Gray | 2.20 | 0.18 | 0.92 |
| Sources: McConkey et al. 2000; McConkey et al. 2013; Campbell et al 2005a. | | | |

**TablE S1. 3. Intercept and slope to measure the harvest index for the major crops**

| **Cropping Activity** | **Intercept:** | **slope:** |
| --- | --- | --- |
| Wheat/Durum | 0.344 | 0.015 |
| Other cereal | 0.380 | 0.015 |
| Barley feed/ barley malt | 0.373 | 0.028 |
| Oats | 0.357 | 0.029 |
| Flax | 0.171 | 0.110 |
| Canola | 0.180 | 0.046 |
| Lentil | 0.305 | 0.059 |
| Field Pea | 0.163 | 0.071 |
| Other Pulse | 0.279 | 0.046 |
| Source: Fan et al 2017 | | |

**Table S1. 4. Nitrogen content of aboveground and belowground residues of major and crops**

| **Crops** | | **N Content of Above-Ground Residues** | **N Content of Below-Ground Residues** | **Ratio of Below-Ground Residues to Harvested Yield** | **Ratio of Above-Ground Residues to Harvested Yield** |
| --- | --- | --- | --- | --- | --- |
| Beans | 0.008 | | 0.008 | 0.81 | 0.19 |
| Grass | 0.015 | | 0.012 | 0.46 | 0.54 |
| Wheat | 0.006 | | 0.009 | 0.72 | 0.28 |
| Barley | 0.007 | | 0.014 | 0.78 | 0.22 |
| Oats | 0.007 | | 0.008 | 0.75 | 0.25 |
| Soybean | 0.008 | | 0.008 | 0.81 | 0.19 |
| Alfalfa | 0.027 | | 0.019 | 0.60 | 0.40 |
| Source: IPCC (2006) | | | | | |
