# Supplementary material for: Contribution of land use practices to GHGs in the Canadian Prairies crop sector
Source: PLoS One. 2021 Dec 17;16(12):e0260946. doi: 10.1371/journal.pone.0260946 (PMC8682883; doi:10.1371/journal.pone.0260946)
Supplement: S2 Table — Annual soil carbon stock (SCS) quantities and values. Annual emissions from fertilizer N application, residue retention, summerfallow, and fuel (crop production and transportation). Annual net GHG balance and values in Alberta, Saskatchewan, and Manitoba. (DOCX) [file pone.0260946.s002.docx]

**S2 Table. Annual estimates of GHG emissions and sinks at the soil-climte zone and provincial levels**

**Table S2.1. Alberta annual soil carbon stock (SCS) quantities and values by provincial and soil zone levels (base year 1985) 1985-2016**

| Year | Alberta soil carbon stock by soil zones  (Mt CO_2_eq) | | | | | Alberta soil carbon stock (Mt CO_2_eq) | Alberta soil carbon stock value (millions in $2018)  CO_2_eq emitting price | | |
| --- | --- | --- | --- | --- | --- | --- | --- | --- | --- |
|  | **Brown** | **Dark Brown** | **Thin Black** | **Thick Black** | **Gray** |  | **$5.00** | **$10.00** | **$15.00** |
| 1985 | (0.017) | (0.015) | (0.007) | (0.005) | (0.003) | (0.048) | 0.120 | 0.241 | 0.361 |
| 1986 | (0.033) | (0.031) | (0.011) | (0.007) | (0.004) | (0.086) | 0.222 | 0.444 | 0.665 |
| 1987 | (0.030) | (0.030) | (0.011) | (0.007) | (0.004) | (0.082) | 0.218 | 0.436 | 0.654 |
| 1988 | (0.021) | (0.031) | (0.012) | (0.008) | (0.005) | (0.076) | 0.208 | 0.416 | 0.623 |
| 1989 | (0.034) | (0.037) | (0.013) | (0.008) | (0.005) | (0.098) | 0.277 | 0.555 | 0.832 |
| 1990 | (0.034) | (0.038) | (0.015) | (0.010) | (0.005) | (0.103) | 0.299 | 0.599 | 0.898 |
| 1991 | (0.059) | (0.055) | (0.019) | (0.014) | (0.012) | (0.159) | 0.485 | 0.970 | 1.455 |
| 1992 | (0.059) | (0.065) | (0.031) | (0.027) | (0.024) | (0.206) | 0.634 | 1.268 | 1.902 |
| 1993 | (0.077) | (0.099) | (0.054) | (0.045) | (0.039) | (0.314) | 0.985 | 1.969 | 2.954 |
| 1994 | (0.071) | (0.115) | (0.070) | (0.063) | (0.055) | (0.373) | 1.171 | 2.343 | 3.514 |
| 1995 | (0.097) | (0.162) | (0.094) | (0.096) | (0.088) | (0.537) | 1.717 | 3.435 | 5.152 |
| 1996 | (0.090) | (0.201) | (0.124) | (0.115) | (0.094) | (0.624) | 2.020 | 4.039 | 6.059 |
| 1997 | (0.109) | (0.244) | (0.162) | (0.148) | (0.102) | (0.766) | 2.517 | 5.034 | 7.552 |
| 1998 | (0.142) | (0.298) | (0.188) | (0.192) | (0.151) | (0.971) | 3.220 | 6.440 | 9.660 |
| 1999 | (0.195) | (0.385) | (0.250) | (0.210) | (0.180) | (1.220) | 4.112 | 8.223 | 12.335 |
| 2000 | (0.148) | (0.384) | (0.295) | (0.321) | (0.300) | (1.448) | 5.002 | 10.005 | 15.007 |
| 2001 | (0.107) | (0.408) | (0.320) | (0.330) | (0.268) | (1.433) | 5.069 | 10.137 | 15.206 |
| 2002 | (0.147) | (0.254) | (0.135) | (0.174) | (0.215) | (0.925) | 3.345 | 6.690 | 10.034 |
| 2003 | (0.217) | (0.535) | (0.354) | (0.389) | (0.310) | (1.805) | 6.708 | 13.417 | 20.125 |
| 2004 | (0.239) | (0.627) | (0.431) | (0.457) | (0.351) | (2.105) | 7.970 | 15.940 | 23.910 |
| 2005 | (0.271) | (0.689) | (0.456) | (0.494) | (0.415) | (2.325) | 9.009 | 18.018 | 27.027 |
| 2006 | (0.263) | (0.679) | (0.443) | (0.449) | (0.375) | (2.210) | 8.741 | 17.481 | 26.222 |
| 2007 | (0.267) | (0.723) | (0.480) | (0.528) | (0.461) | (2.459) | 9.960 | 19.920 | 29.880 |
| 2008 | (0.394) | (1.056) | (0.729) | (0.777) | (0.607) | (3.564) | 14.809 | 29.618 | 44.428 |
| 2009 | (0.357) | (0.942) | (0.682) | (0.714) | (0.705) | (3.400) | 14.172 | 28.344 | 42.517 |
| 2010 | (0.471) | (1.269) | (0.937) | (0.931) | (0.879) | (4.487) | 19.094 | 38.188 | 57.282 |
| 2011 | (0.528) | (1.493) | (1.186) | (1.023) | (1.196) | (5.426) | 23.878 | 47.756 | 71.635 |
| 2012 | (0.619) | (1.533) | (1.111) | (0.942) | (1.256) | (5.461) | 24.464 | 48.928 | 73.392 |
| 2013 | (0.709) | (1.707) | (1.290) | (1.227) | (1.379) | (6.312) | 28.586 | 57.172 | 85.758 |
| 2014 | (0.634) | (1.557) | (1.181) | (1.097) | (1.302) | (5.772) | 26.768 | 53.536 | 80.304 |
| 2015 | (0.547) | (1.506) | (1.158) | (1.099) | (1.301) | (5.610) | 26.382 | 52.763 | 79.145 |
| 2016 | (0.771) | (1.777) | (1.219) | (0.771) | (1.517) | (6.056) | 28.990 | 57.980 | 86.969 |
| Total | **(7.758)** | **(18.947)** | **(13.467)** | **(12.680)** | **(13.610)** | **(66.462)** | **281.153** | **562.305** | **843.458** |

**Table S2.2. Saskatchewan annual soil carbon stock (SCS) quantities and values by provincial and soil zone levels (base year 1985) 1985-2016**

| Year | Saskatchewan soil carbon stock by soil zones  (Mt CO_2_eq) | | | | | Saskatchewan soil carbon stock (Mt CO_2_eq) | Saskatchewan soil carbon stock value (millions in $2018)  CO_2_eq emitting price | | |
| --- | --- | --- | --- | --- | --- | --- | --- | --- | --- |
|  | **Brown** | **Dark Brown** | **Thin Black** | **Thick Black** | **Gray** |  | **$5.00** | **$10.00** | **$15.00** |
| 1985 | (0.05) | (0.12) | (0.04) | (0.03) | (0.01) | (0.26) | 0.66 | 1.32 | 1.98 |
| 1986 | (0.14) | (0.19) | (0.06) | (0.03) | (0.01) | (0.43) | 1.10 | 2.20 | 3.30 |
| 1987 | (0.13) | (0.17) | (0.06) | (0.03) | (0.02) | (0.40) | 1.06 | 2.12 | 3.18 |
| 1988 | (0.06) | (0.17) | (0.06) | (0.04) | (0.02) | (0.21) | 0.58 | 1.16 | 1.75 |
| 1989 | (0.12) | (0.14) | (0.05) | (0.04) | (0.02) | (0.37) | 1.04 | 2.09 | 3.13 |
| 1990 | (0.17) | (0.22) | (0.08) | (0.06) | (0.03) | (0.57) | 1.66 | 3.31 | 4.97 |
| 1991 | (0.21) | (0.22) | (0.08) | (0.08) | (0.05) | (0.64) | 1.97 | 3.93 | 5.90 |
| 1992 | (0.19) | (0.26) | (0.10) | (0.10) | (0.06) | (0.72) | 2.21 | 4.42 | 6.63 |
| 1993 | (0.28) | (0.33) | (0.13) | (0.13) | (0.08) | (0.95) | 2.96 | 5.93 | 8.89 |
| 1994 | (0.30) | (0.40) | (0.16) | (0.17) | (0.11) | (1.14) | 3.59 | 7.18 | 10.77 |
| 1995 | (0.37) | (0.46) | (0.19) | (0.20) | (0.14) | (1.36) | 4.36 | 8.71 | 13.07 |
| 1996 | (0.44) | (0.61) | (0.25) | (0.28) | (0.19) | (1.77) | 5.74 | 11.48 | 17.23 |
| 1997 | (0.41) | (0.66) | (0.35) | (0.45) | (0.31) | (2.18) | 7.18 | 14.35 | 21.53 |
| 1998 | (0.52) | (0.97) | (0.47) | (0.61) | (0.46) | (3.02) | 10.03 | 20.05 | 30.08 |
| 1999 | (0.72) | (1.06) | (0.43) | (0.76) | (0.58) | (3.56) | 12.01 | 24.02 | 36.02 |
| 2000 | (0.84) | (1.15) | (0.53) | (0.77) | (0.60) | (3.90) | 13.47 | 26.94 | 40.41 |
| 2001 | (0.58) | (0.89) | (0.46) | (0.57) | (0.42) | (2.92) | 10.34 | 20.67 | 31.01 |
| 2002 | (0.57) | (0.78) | (0.47) | (0.39) | (0.21) | (2.43) | 8.79 | 17.58 | 26.37 |
| 2003 | (0.67) | (1.07) | (0.54) | (0.81) | (0.61) | (3.70) | 13.76 | 27.51 | 41.27 |
| 2004 | (0.98) | (1.35) | (0.61) | (0.95) | (0.74) | (4.62) | 17.50 | 34.99 | 52.49 |
| 2005 | (1.08) | (1.64) | (0.69) | (1.08) | (0.84) | (5.34) | 20.68 | 41.36 | 62.04 |
| 2006 | (0.98) | (1.69) | (0.83) | (1.12) | (0.86) | (5.48) | 21.66 | 43.33 | 64.99 |
| 2007 | (1.08) | (1.77) | (0.78) | (1.09) | (0.82) | (5.54) | 22.46 | 44.91 | 67.37 |
| 2008 | (1.30) | (2.17) | (1.05) | (1.48) | (1.13) | (7.13) | 29.64 | 59.28 | 88.92 |
| 2009 | (1.33) | (2.32) | (1.14) | (1.58) | (1.15) | (7.52) | 31.34 | 62.68 | 94.02 |
| 2010 | (1.39) | (2.01) | (0.82) | (1.16) | (0.90) | (6.28) | 26.74 | 53.47 | 80.21 |
| 2011 | (1.60) | (2.21) | (0.68) | (1.62) | (1.28) | (7.38) | 32.49 | 64.98 | 97.47 |
| 2012 | (1.70) | (2.48) | (1.00) | (1.43) | (1.12) | (7.73) | 34.62 | 69.23 | 103.85 |
| 2013 | (2.17) | (3.27) | (1.44) | (1.94) | (1.45) | (10.27) | 46.52 | 93.05 | 139.57 |
| 2014 | (1.97) | (2.62) | (1.13) | (1.83) | (1.38) | (8.92) | 41.38 | 82.75 | 124.13 |
| 2015 | (1.81) | (2.76) | (1.31) | (1.98) | (1.47) | (9.33) | 43.85 | 87.71 | 131.56 |
| 2016 | (1.94) | (2.74) | (1.29) | (1.73) | (1.24) | (8.94) | 42.81 | 85.63 | 128.44 |
| Total | **(26.09)** | **(38.90)** | **(17.30)** | **(24.55)** | **(18.34)** | **(125.04)** | **514.18** | **1,028.36** | **1,542.54** |

**Table S2.3. Manitoba annual soil carbon stock (SCS) quantities and values by provincial and soil zone levels (base year 1985) 1985-2016**

| Year | Manitoba soil carbon stock by Soil Zones (Mt CO_2_eq) | | | | Manitoba soil carbon stock (Mt CO_2_eq) | Manitoba soil carbon sequestration value (millions in $2018)  CO_2_eq emitting price | | |
| --- | --- | --- | --- | --- | --- | --- | --- | --- |
|  | Dark Brown | Thin Black | Thick Black | Gray |  | **$5.00** | **$10.00** | **$15.00** |
| 1985 | (0.000) | (0.036) | (0.056) | (0.004) | (0.097) | 0.242 | 0.484 | 0.727 |
| 1986 | (0.000) | (0.040) | (0.056) | (0.003) | (0.100) | 0.256 | 0.513 | 0.769 |
| 1987 | (0.000) | (0.038) | (0.059) | (0.004) | (0.101) | 0.267 | 0.535 | 0.802 |
| 1988 | (0.000) | (0.030) | (0.042) | (0.003) | (0.076) | 0.208 | 0.416 | 0.624 |
| 1989 | (0.000) | (0.038) | (0.074) | (0.005) | (0.117) | 0.331 | 0.661 | 0.992 |
| 1990 | (0.000) | (0.064) | (0.093) | (0.006) | (0.164) | 0.479 | 0.957 | 1.436 |
| 1991 | (0.001) | (0.070) | (0.097) | (0.008) | (0.175) | 0.535 | 1.070 | 1.604 |
| 1992 | (0.001) | (0.101) | (0.127) | (0.008) | (0.237) | 0.730 | 1.460 | 2.190 |
| 1993 | (0.001) | (0.098) | (0.098) | (0.008) | (0.205) | 0.643 | 1.286 | 1.929 |
| 1994 | (0.001) | (0.128) | (0.132) | (0.011) | (0.272) | 0.855 | 1.709 | 2.564 |
| 1995 | (0.001) | (0.134) | (0.132) | (0.011) | (0.279) | 0.891 | 1.782 | 2.673 |
| 1996 | (0.002) | (0.188) | (0.151) | (0.013) | (0.354) | 1.147 | 2.294 | 3.441 |
| 1997 | (0.002) | (0.213) | (0.178) | (0.017) | (0.409) | 1.345 | 2.691 | 4.036 |
| 1998 | (0.003) | (0.304) | (0.252) | (0.023) | (0.581) | 1.927 | 3.854 | 5.782 |
| 1999 | (0.003) | (0.238) | (0.258) | (0.024) | (0.523) | 1.762 | 3.523 | 5.285 |
| 2000 | (0.002) | (0.357) | (0.170) | (0.015) | (0.544) | 1.878 | 3.757 | 5.635 |
| 2001 | (0.002) | (0.332) | (0.135) | (0.012) | (0.481) | 1.700 | 3.401 | 5.101 |
| 2002 | (0.002) | (0.344) | (0.146) | (0.011) | (0.503) | 1.817 | 3.635 | 5.452 |
| 2003 | (0.003) | (0.402) | (0.177) | (0.015) | (0.597) | 2.219 | 4.437 | 6.656 |
| 2004 | (0.002) | (0.423) | (0.158) | (0.012) | (0.595) | 2.251 | 4.503 | 6.754 |
| 2005 | (0.002) | (0.369) | (0.108) | (0.011) | (0.490) | 1.899 | 3.798 | 5.697 |
| 2006 | (0.002) | (0.471) | (0.170) | (0.010) | (0.653) | 2.584 | 5.168 | 7.752 |
| 2007 | (0.003) | (0.488) | (0.191) | (0.018) | (0.701) | 2.838 | 5.676 | 8.514 |
| 2008 | (0.005) | (0.619) | (0.265) | (0.028) | (0.918) | 3.813 | 7.626 | 11.439 |
| 2009 | (0.006) | (0.728) | (0.291) | (0.031) | (1.057) | 4.404 | 8.807 | 13.211 |
| 2010 | (0.006) | (0.654) | (0.276) | (0.031) | (0.966) | 4.113 | 8.226 | 12.339 |
| 2011 | (0.006) | (0.382) | (0.243) | (0.034) | (0.665) | 2.928 | 5.856 | 8.784 |
| 2012 | (0.006) | (0.712) | (0.333) | (0.040) | (1.091) | 4.886 | 9.773 | 14.659 |
| 2013 | (0.009) | (0.809) | (0.413) | (0.052) | (1.283) | 5.809 | 11.619 | 17.428 |
| 2014 | (0.006) | (0.584) | (0.360) | (0.042) | (0.992) | 4.602 | 9.203 | 13.805 |
| 2015 | (0.008) | (0.648) | (0.383) | (0.049) | (1.089) | 5.119 | 10.238 | 15.356 |
| 2016 | (0.007) | (0.634) | (0.401) | (0.044) | (1.086) | 5.200 | 10.400 | 15.600 |
| Total | **(0.094)** | **(10.676)** | **(6.028)** | **(0.602)** | **(17.399)** | **69.679** | **139.357** | **209.036** |

**Table S2.4. Alberta Annual Emission from Fertilizer N Application 1985-2016 (Provincial and soil zone levels)**

| Year | Alberta emission from fertilizer application  (Mt CO_2_eq) | Alberta emission from fertilizer application by soil zone (Mt CO_2_eq) | | | | |
| --- | --- | --- | --- | --- | --- | --- |
|  |  | Brown | Dark Brown | Thin Black | Thick Black | Gray |
| 1985 | 0.988 | 0.044 | 0.182 | 0.227 | 0.212 | 0.323 |
| 1986 | 0.846 | 0.040 | 0.159 | 0.200 | 0.183 | 0.263 |
| 1987 | 0.792 | 0.039 | 0.150 | 0.187 | 0.163 | 0.254 |
| 1988 | 0.864 | 0.040 | 0.164 | 0.206 | 0.180 | 0.274 |
| 1989 | 0.953 | 0.042 | 0.179 | 0.225 | 0.198 | 0.308 |
| 1990 | 0.978 | 0.045 | 0.178 | 0.225 | 0.204 | 0.326 |
| 1991 | 0.929 | 0.048 | 0.175 | 0.221 | 0.188 | 0.297 |
| 1992 | 0.978 | 0.044 | 0.192 | 0.237 | 0.202 | 0.304 |
| 1993 | 1.025 | 0.046 | 0.199 | 0.245 | 0.212 | 0.323 |
| 1994 | 1.078 | 0.051 | 0.215 | 0.256 | 0.224 | 0.332 |
| 1995 | 1.071 | 0.053 | 0.211 | 0.252 | 0.224 | 0.332 |
| 1996 | 1.160 | 0.063 | 0.232 | 0.266 | 0.235 | 0.364 |
| 1997 | 1.161 | 0.066 | 0.248 | 0.290 | 0.247 | 0.310 |
| 1998 | 1.236 | 0.072 | 0.244 | 0.295 | 0.269 | 0.356 |
| 1999 | 1.264 | 0.069 | 0.247 | 0.298 | 0.257 | 0.393 |
| 2000 | 1.305 | 0.070 | 0.271 | 0.305 | 0.264 | 0.396 |
| 2001 | 1.135 | 0.067 | 0.236 | 0.270 | 0.226 | 0.337 |
| 2002 | 1.175 | 0.067 | 0.249 | 0.275 | 0.237 | 0.348 |
| 2003 | 1.061 | 0.062 | 0.213 | 0.238 | 0.217 | 0.331 |
| 2004 | 1.169 | 0.063 | 0.229 | 0.258 | 0.241 | 0.377 |
| 2005 | 1.100 | 0.062 | 0.222 | 0.245 | 0.223 | 0.347 |
| 2006 | 1.078 | 0.065 | 0.224 | 0.246 | 0.220 | 0.324 |
| 2007 | 1.227 | 0.072 | 0.257 | 0.277 | 0.264 | 0.357 |
| 2008 | 1.337 | 0.082 | 0.286 | 0.299 | 0.279 | 0.391 |
| 2009 | 1.316 | 0.070 | 0.265 | 0.288 | 0.274 | 0.419 |
| 2010 | 1.357 | 0.074 | 0.272 | 0.284 | 0.270 | 0.457 |
| 2011 | 1.572 | 0.084 | 0.314 | 0.383 | 0.302 | 0.488 |
| 2012 | 1.651 | 0.091 | 0.315 | 0.336 | 0.402 | 0.507 |
| 2013 | 1.710 | 0.096 | 0.331 | 0.374 | 0.341 | 0.569 |
| 2014 | 1.772 | 0.097 | 0.349 | 0.382 | 0.338 | 0.606 |
| 2015 | 1.816 | 0.101 | 0.360 | 0.387 | 0.351 | 0.617 |
| 2016 | 1.585 | 0.095 | 0.327 | 0.342 | 0.315 | 0.506 |
| Total | **38.688** | **2.079** | **7.693** | **8.818** | **7.962** | **12.136** |

**Table S2.5. Saskatchewan Annual Emission from Fertilizer N Application 1985-2016 (Provincial and soil zone levels)**

| Year | Saskatchewan emission from fertilizer application  (Mt CO_2_eq) | Saskatchewan emission from fertilizer application by soil zone (Mt CO_2_eq) | | | | |
| --- | --- | --- | --- | --- | --- | --- |
|  |  | Brown | Dark Brown | Thin Black | Thick Black | Gray |
| 1985 | 0.76 | 0.07 | 0.15 | 0.13 | 0.24 | 0.17 |
| 1986 | 0.79 | 0.08 | 0.17 | 0.13 | 0.24 | 0.17 |
| 1987 | 0.68 | 0.07 | 0.15 | 0.11 | 0.20 | 0.14 |
| 1988 | 0.67 | 0.07 | 0.14 | 0.11 | 0.21 | 0.15 |
| 1989 | 0.47 | 0.07 | 0.12 | 0.05 | 0.11 | 0.12 |
| 1990 | 0.69 | 0.07 | 0.14 | 0.11 | 0.21 | 0.15 |
| 1991 | 0.62 | 0.06 | 0.13 | 0.10 | 0.20 | 0.14 |
| 1992 | 0.78 | 0.07 | 0.16 | 0.13 | 0.25 | 0.18 |
| 1993 | 0.83 | 0.07 | 0.17 | 0.14 | 0.26 | 0.18 |
| 1994 | 0.99 | 0.09 | 0.21 | 0.17 | 0.31 | 0.21 |
| 1995 | 1.08 | 0.11 | 0.25 | 0.18 | 0.32 | 0.23 |
| 1996 | 1.24 | 0.12 | 0.29 | 0.20 | 0.36 | 0.26 |
| 1997 | 1.43 | 0.14 | 0.33 | 0.24 | 0.42 | 0.30 |
| 1998 | 1.24 | 0.13 | 0.30 | 0.20 | 0.36 | 0.26 |
| 1999 | 1.18 | 0.12 | 0.26 | 0.18 | 0.36 | 0.26 |
| 2000 | 1.27 | 0.13 | 0.29 | 0.21 | 0.37 | 0.27 |
| 2001 | 1.24 | 0.12 | 0.29 | 0.21 | 0.36 | 0.26 |
| 2002 | 1.15 | 0.12 | 0.26 | 0.19 | 0.34 | 0.24 |
| 2003 | 1.31 | 0.14 | 0.29 | 0.21 | 0.39 | 0.28 |
| 2004 | 1.28 | 0.13 | 0.28 | 0.21 | 0.39 | 0.28 |
| 2005 | 1.21 | 0.13 | 0.28 | 0.19 | 0.35 | 0.26 |
| 2006 | 1.12 | 0.12 | 0.28 | 0.20 | 0.30 | 0.22 |
| 2007 | 1.22 | 0.13 | 0.30 | 0.21 | 0.34 | 0.24 |
| 2008 | 1.52 | 0.15 | 0.36 | 0.26 | 0.44 | 0.32 |
| 2009 | 1.47 | 0.14 | 0.33 | 0.25 | 0.44 | 0.30 |
| 2010 | 1.39 | 0.15 | 0.33 | 0.22 | 0.40 | 0.30 |
| 2011 | 1.55 | 0.15 | 0.31 | 0.19 | 0.52 | 0.38 |
| 2012 | 2.00 | 0.19 | 0.46 | 0.32 | 0.59 | 0.44 |
| 2013 | 2.13 | 0.21 | 0.49 | 0.35 | 0.63 | 0.46 |
| 2014 | 2.07 | 0.21 | 0.45 | 0.33 | 0.64 | 0.45 |
| 2015 | 2.23 | 0.22 | 0.49 | 0.36 | 0.68 | 0.48 |
| 2016 | 2.25 | 0.23 | 0.50 | 0.37 | 0.67 | 0.47 |
| Total | **39.88** | **4.01** | **8.97** | **6.46** | **11.89** | **8.56** |

**Table S2.6. Manitoba Annual Emission from Fertilizer N Application 1985-2016 (Provincial and soil zone levels)**

| Year | Manitoba emission from fertilizer application  (Mt CO_2_eq) | Manitoba emission from fertilizer application by soil zone  (Mt CO_2_eq) | | | |  |
| --- | --- | --- | --- | --- | --- | --- |
|  |  | Dark Brown | Thin Black | Thick Black | Gray | |
| 1985 | 0.770 | 0.006 | 0.287 | 0.433 | 0.044 | |
| 1986 | 0.768 | 0.005 | 0.293 | 0.430 | 0.039 | |
| 1987 | 0.726 | 0.005 | 0.277 | 0.408 | 0.036 | |
| 1988 | 0.805 | 0.006 | 0.308 | 0.450 | 0.041 | |
| 1989 | 0.724 | 0.005 | 0.275 | 0.404 | 0.039 | |
| 1990 | 0.759 | 0.006 | 0.288 | 0.425 | 0.041 | |
| 1991 | 0.844 | 0.006 | 0.318 | 0.473 | 0.046 | |
| 1992 | 0.853 | 0.006 | 0.335 | 0.469 | 0.043 | |
| 1993 | 0.927 | 0.007 | 0.352 | 0.516 | 0.051 | |
| 1994 | 0.977 | 0.007 | 0.363 | 0.554 | 0.052 | |
| 1995 | 1.006 | 0.007 | 0.360 | 0.584 | 0.054 | |
| 1996 | 1.009 | 0.008 | 0.374 | 0.573 | 0.054 | |
| 1997 | 1.047 | 0.007 | 0.385 | 0.599 | 0.056 | |
| 1998 | 1.071 | 0.008 | 0.401 | 0.603 | 0.059 | |
| 1999 | 1.055 | 0.008 | 0.364 | 0.624 | 0.059 | |
| 2000 | 1.049 | 0.008 | 0.366 | 0.613 | 0.062 | |
| 2001 | 0.971 | 0.008 | 0.368 | 0.540 | 0.055 | |
| 2002 | 0.989 | 0.008 | 0.376 | 0.548 | 0.056 | |
| 2003 | 1.066 | 0.009 | 0.419 | 0.576 | 0.062 | |
| 2004 | 1.005 | 0.006 | 0.394 | 0.550 | 0.054 | |
| 2005 | 0.845 | 0.008 | 0.348 | 0.445 | 0.044 | |
| 2006 | 0.930 | 0.006 | 0.360 | 0.516 | 0.047 | |
| 2007 | 1.018 | 0.009 | 0.388 | 0.562 | 0.058 | |
| 2008 | 1.092 | 0.009 | 0.407 | 0.611 | 0.065 | |
| 2009 | 0.979 | 0.008 | 0.384 | 0.536 | 0.052 | |
| 2010 | 1.115 | 0.009 | 0.424 | 0.617 | 0.064 | |
| 2011 | 0.993 | 0.009 | 0.314 | 0.608 | 0.062 | |
| 2012 | 1.021 | 0.009 | 0.406 | 0.546 | 0.060 | |
| 2013 | 1.243 | 0.011 | 0.495 | 0.664 | 0.072 | |
| 2014 | 1.186 | 0.010 | 0.445 | 0.659 | 0.072 | |
| 2015 | 1.215 | 0.012 | 0.484 | 0.644 | 0.075 | |
| 2016 | 1.316 | 0.012 | 0.525 | 0.705 | 0.074 | |
| Total | **31.373** | **0.250** | **11.883** | **17.488** | **1.751** | |

**Table S2.7. Alberta Annual Emission from Crop Residue Retention 1985-2016 (provincial and soil zone levels)**

| Year | Alberta emission from crop residue  (Mt CO_2_eq) | Alberta emission from crop residue by soil zone (Mt CO_2_eq) | | | | |
| --- | --- | --- | --- | --- | --- | --- |
|  |  | Brown | Dark Brown | Thin Black | Thick Black | Gray |
| 1985 | 1.067 | 0.095 | 0.269 | 0.196 | 0.219 | 0.288 |
| 1986 | 1.507 | 0.159 | 0.384 | 0.305 | 0.301 | 0.358 |
| 1987 | 1.453 | 0.154 | 0.346 | 0.271 | 0.289 | 0.394 |
| 1988 | 1.466 | 0.101 | 0.322 | 0.247 | 0.299 | 0.497 |
| 1989 | 1.415 | 0.124 | 0.338 | 0.268 | 0.289 | 0.396 |
| 1990 | 1.466 | 0.124 | 0.341 | 0.281 | 0.300 | 0.419 |
| 1991 | 1.427 | 0.166 | 0.354 | 0.271 | 0.283 | 0.353 |
| 1992 | 1.266 | 0.149 | 0.312 | 0.238 | 0.253 | 0.315 |
| 1993 | 1.490 | 0.174 | 0.383 | 0.296 | 0.283 | 0.353 |
| 1994 | 1.461 | 0.164 | 0.350 | 0.277 | 0.295 | 0.376 |
| 1995 | 1.492 | 0.176 | 0.382 | 0.279 | 0.279 | 0.378 |
| 1996 | 1.479 | 0.155 | 0.383 | 0.299 | 0.280 | 0.362 |
| 1997 | 1.311 | 0.141 | 0.373 | 0.279 | 0.248 | 0.270 |
| 1998 | 1.330 | 0.152 | 0.353 | 0.260 | 0.254 | 0.311 |
| 1999 | 1.568 | 0.205 | 0.443 | 0.326 | 0.285 | 0.308 |
| 2000 | 1.401 | 0.145 | 0.383 | 0.268 | 0.254 | 0.351 |
| 2001 | 1.139 | 0.083 | 0.297 | 0.234 | 0.209 | 0.317 |
| 2002 | 0.749 | 0.112 | 0.187 | 0.109 | 0.110 | 0.231 |
| 2003 | 1.382 | 0.170 | 0.381 | 0.269 | 0.239 | 0.323 |
| 2004 | 1.548 | 0.173 | 0.415 | 0.302 | 0.288 | 0.371 |
| 2005 | 1.676 | 0.196 | 0.457 | 0.328 | 0.298 | 0.397 |
| 2006 | 1.551 | 0.189 | 0.437 | 0.303 | 0.270 | 0.352 |
| 2007 | 1.523 | 0.181 | 0.402 | 0.280 | 0.281 | 0.380 |
| 2008 | 1.784 | 0.221 | 0.520 | 0.342 | 0.321 | 0.380 |
| 2009 | 1.380 | 0.172 | 0.390 | 0.254 | 0.236 | 0.328 |
| 2010 | 1.735 | 0.234 | 0.483 | 0.338 | 0.306 | 0.373 |
| 2011 | 1.904 | 0.238 | 0.515 | 0.378 | 0.309 | 0.464 |
| 2012 | 1.879 | 0.253 | 0.494 | 0.331 | 0.346 | 0.455 |
| 2013 | 2.008 | 0.273 | 0.550 | 0.379 | 0.321 | 0.484 |
| 2014 | 1.840 | 0.249 | 0.514 | 0.347 | 0.291 | 0.439 |
| 2015 | 1.685 | 0.201 | 0.481 | 0.325 | 0.270 | 0.409 |
| 2016 | 1.993 | 0.298 | 0.570 | 0.357 | 0.301 | 0.468 |
| Total | **48.376** | **5.629** | **12.807** | **9.237** | **8.807** | **11.896** |

**Table S2.8. Saskatchewan Annual Emission from Crop Residue Retention 1985-2016 (provincial and soil zone levels)**

| Year | Saskatchewan emission from crop residue  (Mt CO_2_eq) | Saskatchewan emission from crop residue by soil zone (Mt CO_2_eq) | | | | |
| --- | --- | --- | --- | --- | --- | --- |
|  |  | Brown | Dark Brown | Thin Black | Thick Black | Gray |
| 1985 | 1.51 | 0.18 | 0.45 | 0.23 | 0.38 | 0.27 |
| 1986 | 1.93 | 0.42 | 0.62 | 0.28 | 0.36 | 0.25 |
| 1987 | 1.71 | 0.37 | 0.54 | 0.24 | 0.33 | 0.23 |
| 1988 | 1.02 | 0.16 | 0.26 | 0.16 | 0.26 | 0.18 |
| 1989 | 1.52 | 0.34 | 0.40 | 0.19 | 0.35 | 0.26 |
| 1990 | 1.95 | 0.37 | 0.59 | 0.28 | 0.41 | 0.30 |
| 1991 | 1.90 | 0.41 | 0.56 | 0.25 | 0.39 | 0.29 |
| 1992 | 1.70 | 0.35 | 0.55 | 0.24 | 0.32 | 0.23 |
| 1993 | 1.87 | 0.42 | 0.58 | 0.25 | 0.35 | 0.26 |
| 1994 | 1.93 | 0.40 | 0.60 | 0.26 | 0.39 | 0.29 |
| 1995 | 1.82 | 0.41 | 0.56 | 0.24 | 0.35 | 0.26 |
| 1996 | 2.10 | 0.45 | 0.66 | 0.28 | 0.41 | 0.30 |
| 1997 | 1.83 | 0.39 | 0.56 | 0.25 | 0.37 | 0.27 |
| 1998 | 1.99 | 0.39 | 0.62 | 0.28 | 0.40 | 0.30 |
| 1999 | 2.31 | 0.49 | 0.72 | 0.28 | 0.47 | 0.35 |
| 2000 | 2.31 | 0.51 | 0.70 | 0.31 | 0.45 | 0.34 |
| 2001 | 1.66 | 0.33 | 0.50 | 0.25 | 0.34 | 0.24 |
| 2002 | 1.31 | 0.31 | 0.42 | 0.24 | 0.22 | 0.12 |
| 2003 | 1.81 | 0.35 | 0.54 | 0.26 | 0.38 | 0.28 |
| 2004 | 2.19 | 0.51 | 0.67 | 0.28 | 0.41 | 0.31 |
| 2005 | 2.36 | 0.54 | 0.75 | 0.30 | 0.43 | 0.33 |
| 2006 | 2.11 | 0.42 | 0.70 | 0.31 | 0.39 | 0.29 |
| 2007 | 2.00 | 0.42 | 0.67 | 0.28 | 0.36 | 0.27 |
| 2008 | 2.34 | 0.47 | 0.75 | 0.33 | 0.46 | 0.34 |
| 2009 | 2.41 | 0.47 | 0.78 | 0.35 | 0.47 | 0.33 |
| 2010 | 2.06 | 0.52 | 0.68 | 0.26 | 0.34 | 0.25 |
| 2011 | 2.29 | 0.55 | 0.71 | 0.22 | 0.46 | 0.35 |
| 2012 | 2.34 | 0.57 | 0.76 | 0.30 | 0.41 | 0.31 |
| 2013 | 3.03 | 0.72 | 0.99 | 0.41 | 0.53 | 0.39 |
| 2014 | 2.62 | 0.65 | 0.80 | 0.32 | 0.48 | 0.36 |
| 2015 | 2.66 | 0.57 | 0.83 | 0.37 | 0.52 | 0.38 |
| 2016 | 2.73 | 0.67 | 0.87 | 0.38 | 0.47 | 0.34 |
| Total | **65.31** | **14.13** | **20.39** | **8.86** | **12.65** | **9.28** |

**Table S2.9. Manitoba Annual Emission from Crop Residue Retention 1985-2016 (provincial and soil zone levels)**

| Year | Manitoba emission from crop residue (Mt CO_2_eq) | Manitoba emission from crop residue by soil zone (Mt CO_2_eq) | | | |
| --- | --- | --- | --- | --- | --- |
|  |  | Dark Brown | Thin Black | Thick Black | Gray |
| 1985 | 0.739 | 0.006 | 0.274 | 0.411 | 0.047 |
| 1986 | 0.705 | 0.006 | 0.272 | 0.384 | 0.044 |
| 1987 | 0.642 | 0.005 | 0.234 | 0.363 | 0.040 |
| 1988 | 0.446 | 0.005 | 0.171 | 0.237 | 0.033 |
| 1989 | 0.602 | 0.005 | 0.191 | 0.362 | 0.044 |
| 1990 | 0.774 | 0.006 | 0.296 | 0.421 | 0.050 |
| 1991 | 0.675 | 0.006 | 0.262 | 0.361 | 0.046 |
| 1992 | 0.742 | 0.006 | 0.281 | 0.410 | 0.045 |
| 1993 | 0.591 | 0.005 | 0.234 | 0.312 | 0.040 |
| 1994 | 0.680 | 0.006 | 0.248 | 0.381 | 0.045 |
| 1995 | 0.597 | 0.005 | 0.207 | 0.345 | 0.040 |
| 1996 | 0.713 | 0.006 | 0.260 | 0.400 | 0.047 |
| 1997 | 0.618 | 0.005 | 0.222 | 0.350 | 0.040 |
| 1998 | 0.708 | 0.006 | 0.259 | 0.397 | 0.046 |
| 1999 | 0.672 | 0.006 | 0.215 | 0.402 | 0.048 |
| 2000 | 0.761 | 0.007 | 0.294 | 0.410 | 0.050 |
| 2001 | 0.655 | 0.006 | 0.262 | 0.341 | 0.045 |
| 2002 | 0.668 | 0.006 | 0.248 | 0.371 | 0.044 |
| 2003 | 0.740 | 0.007 | 0.274 | 0.413 | 0.047 |
| 2004 | 0.701 | 0.006 | 0.274 | 0.374 | 0.047 |
| 2005 | 0.536 | 0.007 | 0.236 | 0.253 | 0.040 |
| 2006 | 0.744 | 0.006 | 0.277 | 0.414 | 0.047 |
| 2007 | 0.714 | 0.006 | 0.269 | 0.391 | 0.047 |
| 2008 | 0.828 | 0.007 | 0.314 | 0.454 | 0.052 |
| 2009 | 0.797 | 0.007 | 0.324 | 0.421 | 0.045 |
| 2010 | 0.705 | 0.006 | 0.278 | 0.379 | 0.042 |
| 2011 | 0.554 | 0.006 | 0.178 | 0.330 | 0.040 |
| 2012 | 0.755 | 0.005 | 0.298 | 0.406 | 0.045 |
| 2013 | 0.890 | 0.008 | 0.342 | 0.485 | 0.055 |
| 2014 | 0.767 | 0.006 | 0.272 | 0.440 | 0.048 |
| 2015 | 0.840 | 0.008 | 0.318 | 0.457 | 0.057 |
| 2016 | 0.889 | 0.007 | 0.335 | 0.490 | 0.055 |
| Total | **22.448** | **0.195** | **8.420** | **12.368** | **1.464** |

**Table S2.10. Alberta Annual Emission from Summerfallow 1985-2016 (provincial and soil zone levels)**

| Year | Alberta emission from summerfallow  (Mt CO_2_eq) | Alberta emission from summerfallow by soil zone (Mt CO_2_eq) | | | | |
| --- | --- | --- | --- | --- | --- | --- |
|  |  | Brown | Dark Brown | Thin Black | Thick Black | Gray |
| 1985 | 0.395 | 0.087 | 0.133 | 0.056 | 0.032 | 0.087 |
| 1986 | 0.428 | 0.087 | 0.139 | 0.061 | 0.036 | 0.106 |
| 1987 | 0.512 | 0.120 | 0.172 | 0.079 | 0.050 | 0.092 |
| 1988 | 0.481 | 0.122 | 0.164 | 0.069 | 0.041 | 0.085 |
| 1989 | 0.414 | 0.085 | 0.146 | 0.061 | 0.035 | 0.088 |
| 1990 | 0.453 | 0.101 | 0.163 | 0.071 | 0.040 | 0.078 |
| 1991 | 0.421 | 0.099 | 0.150 | 0.054 | 0.036 | 0.083 |
| 1992 | 0.386 | 0.126 | 0.143 | 0.041 | 0.024 | 0.052 |
| 1993 | 0.354 | 0.111 | 0.134 | 0.039 | 0.019 | 0.051 |
| 1994 | 0.359 | 0.111 | 0.129 | 0.038 | 0.020 | 0.061 |
| 1995 | 0.366 | 0.113 | 0.125 | 0.040 | 0.027 | 0.060 |
| 1996 | 0.346 | 0.119 | 0.127 | 0.035 | 0.015 | 0.048 |
| 1997 | 0.362 | 0.099 | 0.111 | 0.041 | 0.019 | 0.092 |
| 1998 | 0.319 | 0.103 | 0.124 | 0.034 | 0.016 | 0.042 |
| 1999 | 0.284 | 0.098 | 0.100 | 0.030 | 0.019 | 0.039 |
| 2000 | 0.292 | 0.107 | 0.092 | 0.032 | 0.017 | 0.045 |
| 2001 | 0.349 | 0.108 | 0.112 | 0.033 | 0.022 | 0.074 |
| 2002 | 0.214 | 0.069 | 0.079 | 0.024 | 0.011 | 0.031 |
| 2003 | 0.168 | 0.064 | 0.058 | 0.013 | 0.007 | 0.026 |
| 2004 | 0.205 | 0.087 | 0.073 | 0.016 | 0.008 | 0.021 |
| 2005 | 0.229 | 0.078 | 0.076 | 0.020 | 0.015 | 0.040 |
| 2006 | 0.252 | 0.089 | 0.080 | 0.024 | 0.016 | 0.044 |
| 2007 | 0.229 | 0.078 | 0.064 | 0.020 | 0.015 | 0.051 |
| 2008 | 0.174 | 0.074 | 0.046 | 0.015 | 0.009 | 0.029 |
| 2009 | 0.213 | 0.103 | 0.058 | 0.015 | 0.011 | 0.026 |
| 2010 | 0.179 | 0.073 | 0.052 | 0.019 | 0.011 | 0.024 |
| 2011 | 0.182 | 0.083 | 0.051 | 0.015 | 0.011 | 0.022 |
| 2012 | 0.155 | 0.079 | 0.045 | 0.010 | 0.004 | 0.016 |
| 2013 | 0.121 | 0.051 | 0.027 | 0.007 | 0.008 | 0.027 |
| 2014 | 0.116 | 0.053 | 0.026 | 0.011 | 0.006 | 0.019 |
| 2015 | 0.095 | 0.051 | 0.025 | 0.006 | 0.003 | 0.010 |
| 2016 | 0.071 | 0.029 | 0.017 | 0.006 | 0.004 | 0.015 |
| Total | **9.125** | **2.856** | **3.040** | **1.036** | **0.609** | **1.584** |

**Table S2.11. Saskatchewan Annual Emission from Summerfallow 1985-2016 (provincial and soil zone levels)**

| Year | Saskatchewan emission from summerfallow  (Mt CO_2_eq) | Saskatchewan emission from summerfallow by soil zone (Mt CO_2_eq) | | | | |
| --- | --- | --- | --- | --- | --- | --- |
|  |  | Brown | Dark Brown | Thin Black | Thick Black | Gray |
| 1985 | 0.88 | 0.25 | 0.30 | 0.12 | 0.13 | 0.08 |
| 1986 | 0.86 | 0.18 | 0.30 | 0.13 | 0.15 | 0.10 |
| 1987 | 1.26 | 0.37 | 0.44 | 0.17 | 0.17 | 0.11 |
| 1988 | 1.17 | 0.37 | 0.41 | 0.15 | 0.15 | 0.10 |
| 1989 | 0.68 | 0.17 | 0.22 | 0.11 | 0.12 | 0.07 |
| 1990 | 0.91 | 0.30 | 0.29 | 0.11 | 0.13 | 0.08 |
| 1991 | 1.09 | 0.33 | 0.39 | 0.15 | 0.14 | 0.09 |
| 1992 | 1.05 | 0.40 | 0.35 | 0.12 | 0.11 | 0.07 |
| 1993 | 0.93 | 0.31 | 0.34 | 0.11 | 0.10 | 0.07 |
| 1994 | 0.94 | 0.34 | 0.30 | 0.11 | 0.11 | 0.07 |
| 1995 | 0.98 | 0.34 | 0.31 | 0.12 | 0.13 | 0.08 |
| 1996 | 0.90 | 0.33 | 0.29 | 0.10 | 0.11 | 0.07 |
| 1997 | 1.01 | 0.34 | 0.22 | 0.11 | 0.20 | 0.15 |
| 1998 | 0.81 | 0.32 | 0.26 | 0.09 | 0.09 | 0.06 |
| 1999 | 0.93 | 0.31 | 0.34 | 0.14 | 0.08 | 0.05 |
| 2000 | 0.76 | 0.31 | 0.24 | 0.07 | 0.08 | 0.05 |
| 2001 | 0.71 | 0.28 | 0.22 | 0.08 | 0.08 | 0.05 |
| 2002 | 0.53 | 0.22 | 0.18 | 0.06 | 0.05 | 0.03 |
| 2003 | 0.40 | 0.16 | 0.13 | 0.05 | 0.04 | 0.02 |
| 2004 | 0.46 | 0.20 | 0.15 | 0.05 | 0.04 | 0.03 |
| 2005 | 0.58 | 0.21 | 0.18 | 0.07 | 0.08 | 0.05 |
| 2006 | 0.81 | 0.25 | 0.21 | 0.09 | 0.15 | 0.10 |
| 2007 | 0.45 | 0.16 | 0.13 | 0.05 | 0.06 | 0.04 |
| 2008 | 0.34 | 0.16 | 0.11 | 0.03 | 0.03 | 0.02 |
| 2009 | 0.40 | 0.18 | 0.13 | 0.04 | 0.03 | 0.02 |
| 2010 | 1.21 | 0.18 | 0.32 | 0.18 | 0.32 | 0.21 |
| 2011 | 1.31 | 0.21 | 0.51 | 0.50 | 0.07 | 0.03 |
| 2012 | 0.31 | 0.11 | 0.08 | 0.03 | 0.05 | 0.02 |
| 2013 | 0.27 | 0.11 | 0.08 | 0.03 | 0.03 | 0.02 |
| 2014 | 0.35 | 0.09 | 0.11 | 0.07 | 0.05 | 0.03 |
| 2015 | 0.18 | 0.07 | 0.06 | 0.02 | 0.02 | 0.01 |
| 2016 | 0.19 | 0.06 | 0.06 | 0.02 | 0.03 | 0.02 |
| Total | **23.67** | **7.60** | **7.67** | **3.28** | **3.11** | **2.00** |

**Table S2.12. Manitoba Annual Emission from Summerfallow 1985-2016 (provincial and soil zone levels)**

| Year | Manitoba emission from summerfallow  (Mt CO_2_eq) | Manitoba emission from summerfallow by soil zone (Mt CO_2_eq) | | | |
| --- | --- | --- | --- | --- | --- |
|  |  | Dark Brown | Thin Black | Thick Black | Gray |
| 1985 | 0.097 | 0.001 | 0.047 | 0.040 | 0.009 |
| 1986 | 0.149 | 0.002 | 0.062 | 0.070 | 0.016 |
| 1987 | 0.150 | 0.002 | 0.067 | 0.068 | 0.013 |
| 1988 | 0.112 | 0.002 | 0.052 | 0.050 | 0.010 |
| 1989 | 0.082 | 0.001 | 0.040 | 0.034 | 0.007 |
| 1990 | 0.082 | 0.001 | 0.039 | 0.034 | 0.008 |
| 1991 | 0.085 | 0.001 | 0.043 | 0.034 | 0.007 |
| 1992 | 0.065 | 0.001 | 0.031 | 0.027 | 0.006 |
| 1993 | 0.068 | 0.001 | 0.034 | 0.028 | 0.005 |
| 1994 | 0.065 | 0.001 | 0.031 | 0.027 | 0.006 |
| 1995 | 0.093 | 0.001 | 0.046 | 0.039 | 0.007 |
| 1996 | 0.094 | 0.001 | 0.041 | 0.043 | 0.009 |
| 1997 | 0.070 | 0.001 | 0.030 | 0.034 | 0.005 |
| 1998 | 0.054 | 0.001 | 0.025 | 0.023 | 0.004 |
| 1999 | 0.235 | 0.001 | 0.171 | 0.055 | 0.008 |
| 2000 | 0.046 | 0.001 | 0.020 | 0.021 | 0.004 |
| 2001 | 0.089 | 0.001 | 0.032 | 0.050 | 0.006 |
| 2002 | 0.037 | 0.000 | 0.020 | 0.014 | 0.003 |
| 2003 | 0.027 | 0.000 | 0.014 | 0.010 | 0.002 |
| 2004 | 0.076 | 0.001 | 0.033 | 0.037 | 0.005 |
| 2005 | 0.217 | 0.001 | 0.040 | 0.157 | 0.019 |
| 2006 | 0.035 | 0.000 | 0.019 | 0.013 | 0.003 |
| 2007 | 0.035 | 0.000 | 0.015 | 0.017 | 0.003 |
| 2008 | 0.020 | 0.000 | 0.010 | 0.009 | 0.001 |
| 2009 | 0.072 | 0.000 | 0.016 | 0.044 | 0.011 |
| 2010 | 0.104 | 0.002 | 0.051 | 0.040 | 0.011 |
| 2011 | 0.532 | 0.003 | 0.342 | 0.165 | 0.022 |
| 2012 | 0.017 | 0.000 | 0.005 | 0.010 | 0.002 |
| 2013 | 0.030 | 0.000 | 0.017 | 0.011 | 0.002 |
| 2014 | 0.208 | 0.002 | 0.150 | 0.045 | 0.011 |
| 2015 | 0.025 | 0.000 | 0.011 | 0.011 | 0.003 |
| 2016 | 0.016 | 0.000 | 0.006 | 0.008 | 0.002 |
| Total | 3.088 | 0.031 | 1.559 | 1.268 | 0.230 |

**Table S2.13. Alberta Annual Emission from Total Fuel Uses 1985-2016 (provincial and soil zone levels)**

| Year | Alberta emission from fuel used (Mt CO_2_eq) | Alberta emission from fuel by soil zone (Mt CO_2_eq) | | | | |
| --- | --- | --- | --- | --- | --- | --- |
|  |  | Brown | Dark Brown | Thin Black | Thick Black | Gray |
| 1985 | 1.263 | 0.181 | 0.340 | 0.252 | 0.199 | 0.292 |
| 1986 | 1.407 | 0.204 | 0.387 | 0.286 | 0.218 | 0.312 |
| 1987 | 1.363 | 0.197 | 0.374 | 0.277 | 0.208 | 0.307 |
| 1988 | 1.329 | 0.185 | 0.362 | 0.271 | 0.209 | 0.302 |
| 1989 | 1.535 | 0.221 | 0.421 | 0.315 | 0.235 | 0.343 |
| 1990 | 1.507 | 0.212 | 0.405 | 0.305 | 0.233 | 0.352 |
| 1991 | 1.492 | 0.216 | 0.411 | 0.301 | 0.227 | 0.338 |
| 1992 | 1.454 | 0.208 | 0.411 | 0.295 | 0.222 | 0.318 |
| 1993 | 1.521 | 0.215 | 0.429 | 0.311 | 0.232 | 0.334 |
| 1994 | 1.463 | 0.202 | 0.422 | 0.298 | 0.223 | 0.318 |
| 1995 | 1.602 | 0.232 | 0.454 | 0.320 | 0.247 | 0.349 |
| 1996 | 1.847 | 0.262 | 0.538 | 0.367 | 0.275 | 0.404 |
| 1997 | 1.792 | 0.259 | 0.541 | 0.381 | 0.273 | 0.337 |
| 1998 | 1.941 | 0.278 | 0.556 | 0.399 | 0.300 | 0.408 |
| 1999 | 1.977 | 0.284 | 0.573 | 0.409 | 0.294 | 0.418 |
| 2000 | 1.944 | 0.283 | 0.572 | 0.388 | 0.285 | 0.416 |
| 2001 | 1.729 | 0.244 | 0.508 | 0.355 | 0.250 | 0.372 |
| 2002 | 1.670 | 0.254 | 0.486 | 0.335 | 0.237 | 0.359 |
| 2003 | 1.777 | 0.271 | 0.512 | 0.346 | 0.259 | 0.389 |
| 2004 | 1.782 | 0.252 | 0.509 | 0.352 | 0.269 | 0.399 |
| 2005 | 1.770 | 0.254 | 0.518 | 0.348 | 0.263 | 0.387 |
| 2006 | 1.902 | 0.283 | 0.568 | 0.374 | 0.278 | 0.399 |
| 2007 | 1.860 | 0.273 | 0.559 | 0.359 | 0.285 | 0.383 |
| 2008 | 1.981 | 0.302 | 0.598 | 0.379 | 0.298 | 0.403 |
| 2009 | 1.888 | 0.260 | 0.552 | 0.366 | 0.290 | 0.420 |
| 2010 | 1.680 | 0.242 | 0.497 | 0.313 | 0.246 | 0.382 |
| 2011 | 2.022 | 0.264 | 0.576 | 0.451 | 0.293 | 0.439 |
| 2012 | 2.101 | 0.304 | 0.595 | 0.376 | 0.370 | 0.455 |
| 2013 | 2.038 | 0.308 | 0.583 | 0.388 | 0.296 | 0.463 |
| 2014 | 2.201 | 0.328 | 0.627 | 0.413 | 0.309 | 0.524 |
| 2015 | 2.229 | 0.335 | 0.632 | 0.415 | 0.314 | 0.532 |
| 2016 | 2.371 | 0.371 | 0.705 | 0.442 | 0.332 | 0.522 |
| Total | **56.439** | **8.183** | **16.224** | **11.188** | **8.469** | **12.375** |

**Table S2.14. Saskatchewan Annual Emission from Total Fuel Uses 1985-2016 (provincial and soil zone levels)**

| Year | Saskatchewan emission from fuel used (Mt CO_2_eq) | Saskatchewan emission from fuel by soil zone (Mt CO_2_eq) | | | | |
| --- | --- | --- | --- | --- | --- | --- |
|  |  | Brown | Dark Brown | Thin Black | Thick Black | Gray |
| 1985 | 1.98 | 0.46 | 0.63 | 0.28 | 0.37 | 0.26 |
| 1986 | 2.06 | 0.50 | 0.66 | 0.28 | 0.36 | 0.25 |
| 1987 | 2.00 | 0.49 | 0.64 | 0.27 | 0.35 | 0.25 |
| 1988 | 2.09 | 0.49 | 0.65 | 0.29 | 0.39 | 0.27 |
| 1989 | 2.36 | 0.57 | 0.72 | 0.31 | 0.44 | 0.31 |
| 1990 | 2.42 | 0.56 | 0.75 | 0.33 | 0.45 | 0.32 |
| 1991 | 2.12 | 0.50 | 0.66 | 0.29 | 0.39 | 0.27 |
| 1992 | 2.00 | 0.46 | 0.65 | 0.28 | 0.36 | 0.25 |
| 1993 | 2.07 | 0.51 | 0.66 | 0.28 | 0.37 | 0.26 |
| 1994 | 2.21 | 0.54 | 0.70 | 0.30 | 0.40 | 0.28 |
| 1995 | 2.31 | 0.58 | 0.73 | 0.30 | 0.41 | 0.29 |
| 1996 | 2.51 | 0.61 | 0.80 | 0.33 | 0.44 | 0.32 |
| 1997 | 2.57 | 0.61 | 0.79 | 0.34 | 0.48 | 0.34 |
| 1998 | 2.29 | 0.55 | 0.74 | 0.30 | 0.40 | 0.29 |
| 1999 | 2.30 | 0.56 | 0.74 | 0.29 | 0.41 | 0.29 |
| 2000 | 2.31 | 0.55 | 0.75 | 0.31 | 0.41 | 0.29 |
| 2001 | 1.96 | 0.48 | 0.63 | 0.27 | 0.34 | 0.24 |
| 2002 | 1.85 | 0.46 | 0.60 | 0.26 | 0.31 | 0.21 |
| 2003 | 1.85 | 0.45 | 0.59 | 0.25 | 0.33 | 0.24 |
| 2004 | 1.88 | 0.46 | 0.60 | 0.24 | 0.33 | 0.24 |
| 2005 | 2.07 | 0.53 | 0.68 | 0.26 | 0.34 | 0.25 |
| 2006 | 2.08 | 0.51 | 0.70 | 0.29 | 0.34 | 0.24 |
| 2007 | 2.43 | 0.62 | 0.83 | 0.31 | 0.39 | 0.28 |
| 2008 | 2.53 | 0.62 | 0.84 | 0.34 | 0.43 | 0.31 |
| 2009 | 2.48 | 0.60 | 0.82 | 0.34 | 0.43 | 0.30 |
| 2010 | 2.60 | 0.68 | 0.87 | 0.32 | 0.42 | 0.31 |
| 2011 | 2.68 | 0.69 | 0.85 | 0.27 | 0.49 | 0.37 |
| 2012 | 2.91 | 0.74 | 0.96 | 0.37 | 0.48 | 0.35 |
| 2013 | 3.26 | 0.82 | 1.08 | 0.43 | 0.54 | 0.39 |
| 2014 | 3.89 | 1.02 | 1.22 | 0.47 | 0.68 | 0.50 |
| 2015 | 4.13 | 1.00 | 1.32 | 0.54 | 0.73 | 0.53 |
| 2016 | 3.90 | 1.02 | 1.29 | 0.52 | 0.63 | 0.45 |
| Total | 78.07 | 19.25 | 25.16 | 10.29 | 13.61 | 9.76 |

**Table S2.15. Manitoba Annual Emission from Total Fuel 1985-2016 (provincial and soil zone levels)**

| Year | Manitoba emission from fuel used (Mt CO_2_eq) | Manitoba emission from fuel by soil zone (Mt CO_2_eq) | | | |
| --- | --- | --- | --- | --- | --- |
|  |  | Dark Brown | Thin Black | Thick Black | Gray |
| 1985 | 0.626 | 0.006 | 0.253 | 0.329 | 0.039 |
| 1986 | 0.610 | 0.005 | 0.252 | 0.318 | 0.035 |
| 1987 | 0.592 | 0.005 | 0.241 | 0.312 | 0.035 |
| 1988 | 0.623 | 0.005 | 0.255 | 0.325 | 0.037 |
| 1989 | 0.661 | 0.006 | 0.263 | 0.353 | 0.039 |
| 1990 | 0.671 | 0.006 | 0.274 | 0.353 | 0.039 |
| 1991 | 0.707 | 0.006 | 0.292 | 0.369 | 0.041 |
| 1992 | 0.721 | 0.005 | 0.301 | 0.376 | 0.038 |
| 1993 | 0.698 | 0.006 | 0.288 | 0.364 | 0.040 |
| 1994 | 0.700 | 0.006 | 0.279 | 0.376 | 0.040 |
| 1995 | 0.676 | 0.006 | 0.259 | 0.372 | 0.039 |
| 1996 | 0.706 | 0.006 | 0.276 | 0.383 | 0.041 |
| 1997 | 0.684 | 0.005 | 0.265 | 0.374 | 0.039 |
| 1998 | 0.683 | 0.005 | 0.268 | 0.370 | 0.039 |
| 1999 | 0.651 | 0.006 | 0.246 | 0.362 | 0.037 |
| 2000 | 0.726 | 0.006 | 0.286 | 0.392 | 0.042 |
| 2001 | 0.730 | 0.006 | 0.287 | 0.394 | 0.043 |
| 2002 | 0.730 | 0.006 | 0.282 | 0.400 | 0.042 |
| 2003 | 0.742 | 0.006 | 0.295 | 0.399 | 0.043 |
| 2004 | 0.792 | 0.005 | 0.321 | 0.424 | 0.042 |
| 2005 | 0.719 | 0.007 | 0.300 | 0.374 | 0.039 |
| 2006 | 0.793 | 0.005 | 0.309 | 0.438 | 0.041 |
| 2007 | 0.886 | 0.007 | 0.349 | 0.480 | 0.050 |
| 2008 | 0.919 | 0.008 | 0.355 | 0.503 | 0.053 |
| 2009 | 0.783 | 0.006 | 0.314 | 0.422 | 0.040 |
| 2010 | 0.755 | 0.006 | 0.295 | 0.410 | 0.044 |
| 2011 | 0.713 | 0.006 | 0.239 | 0.423 | 0.045 |
| 2012 | 0.803 | 0.006 | 0.315 | 0.435 | 0.047 |
| 2013 | 0.851 | 0.007 | 0.329 | 0.465 | 0.050 |
| 2014 | 0.893 | 0.007 | 0.324 | 0.509 | 0.052 |
| 2015 | 0.947 | 0.009 | 0.364 | 0.514 | 0.060 |
| 2016 | 0.968 | 0.008 | 0.369 | 0.536 | 0.056 |
| Total | 23.758 | 0.194 | 9.346 | 12.852 | 1.366 |

**Table S2.16. Alberta Annual Emission from Fuel on Farm for Crop Production 1985-2016 (provincial and soil zone levels)**

| Year | Alberta aggerate emission from fuel on farm (Mt CO_2_eq) | Alberta emission from fuel on farm by soil zone (Mt CO_2_eq) | | | | |
| --- | --- | --- | --- | --- | --- | --- |
|  |  | Brown | Dark Brown | Thin Black | Thick Black | Gray |
| 1985 | 1.061 | 0.159 | 0.285 | 0.212 | 0.161 | 0.244 |
| 1986 | 1.054 | 0.160 | 0.287 | 0.209 | 0.158 | 0.240 |
| 1987 | 1.038 | 0.157 | 0.284 | 0.208 | 0.153 | 0.234 |
| 1988 | 1.013 | 0.154 | 0.279 | 0.206 | 0.151 | 0.222 |
| 1989 | 1.023 | 0.158 | 0.281 | 0.207 | 0.151 | 0.226 |
| 1990 | 0.996 | 0.151 | 0.269 | 0.198 | 0.148 | 0.230 |
| 1991 | 0.982 | 0.146 | 0.271 | 0.196 | 0.145 | 0.224 |
| 1992 | 0.971 | 0.144 | 0.276 | 0.196 | 0.143 | 0.212 |
| 1993 | 0.983 | 0.146 | 0.277 | 0.197 | 0.145 | 0.217 |
| 1994 | 0.957 | 0.138 | 0.278 | 0.192 | 0.141 | 0.208 |
| 1995 | 0.958 | 0.145 | 0.272 | 0.190 | 0.144 | 0.207 |
| 1996 | 0.960 | 0.146 | 0.279 | 0.187 | 0.137 | 0.210 |
| 1997 | 0.934 | 0.142 | 0.280 | 0.194 | 0.137 | 0.180 |
| 1998 | 0.917 | 0.138 | 0.263 | 0.186 | 0.137 | 0.193 |
| 1999 | 0.906 | 0.135 | 0.261 | 0.184 | 0.132 | 0.195 |
| 2000 | 0.896 | 0.140 | 0.264 | 0.177 | 0.126 | 0.189 |
| 2001 | 0.804 | 0.124 | 0.237 | 0.161 | 0.110 | 0.172 |
| 2002 | 0.799 | 0.126 | 0.233 | 0.161 | 0.111 | 0.168 |
| 2003 | 0.798 | 0.128 | 0.228 | 0.154 | 0.112 | 0.175 |
| 2004 | 0.780 | 0.116 | 0.221 | 0.152 | 0.113 | 0.178 |
| 2005 | 0.766 | 0.114 | 0.223 | 0.149 | 0.111 | 0.169 |
| 2006 | 0.765 | 0.117 | 0.226 | 0.149 | 0.110 | 0.163 |
| 2007 | 0.746 | 0.114 | 0.222 | 0.143 | 0.112 | 0.155 |
| 2008 | 0.758 | 0.121 | 0.226 | 0.143 | 0.110 | 0.158 |
| 2009 | 0.731 | 0.105 | 0.214 | 0.140 | 0.109 | 0.163 |
| 2010 | 0.680 | 0.104 | 0.200 | 0.124 | 0.095 | 0.157 |
| 2011 | 0.729 | 0.101 | 0.207 | 0.162 | 0.102 | 0.157 |
| 2012 | 0.770 | 0.117 | 0.217 | 0.136 | 0.138 | 0.163 |
| 2013 | 0.717 | 0.112 | 0.206 | 0.135 | 0.100 | 0.163 |
| 2014 | 0.731 | 0.113 | 0.209 | 0.136 | 0.099 | 0.175 |
| 2015 | 0.744 | 0.118 | 0.212 | 0.137 | 0.101 | 0.177 |
| 2016 | 0.760 | 0.120 | 0.226 | 0.142 | 0.110 | 0.162 |
| Total | **27.725** | **4.211** | **7.915** | **5.462** | **4.049** | **6.088** |

**Table S2.17. Saskatchewan Annual Emission from Fuel on Farm for Crop Production 1985-2016 (provincial and soil zone levels)**

| Year | Saskatchewan aggerate emission from fuel on farm (Mt CO_2_eq) | Saskatchewan emission from fuel on farm by soil zone (Mt CO_2_eq) | | | | |
| --- | --- | --- | --- | --- | --- | --- |
|  |  | Brown | Dark Brown | Thin Black | Thick Black | Gray |
| 1985 | 1.84 | 0.44 | 0.58 | 0.25 | 0.33 | 0.23 |
| 1986 | 1.86 | 0.46 | 0.59 | 0.25 | 0.33 | 0.23 |
| 1987 | 1.83 | 0.45 | 0.58 | 0.25 | 0.32 | 0.22 |
| 1988 | 1.82 | 0.43 | 0.57 | 0.25 | 0.33 | 0.23 |
| 1989 | 1.87 | 0.46 | 0.58 | 0.25 | 0.34 | 0.24 |
| 1990 | 1.84 | 0.44 | 0.57 | 0.25 | 0.34 | 0.24 |
| 1991 | 1.81 | 0.43 | 0.57 | 0.25 | 0.33 | 0.23 |
| 1992 | 1.73 | 0.40 | 0.56 | 0.24 | 0.31 | 0.22 |
| 1993 | 1.77 | 0.44 | 0.56 | 0.24 | 0.31 | 0.22 |
| 1994 | 1.79 | 0.44 | 0.56 | 0.24 | 0.32 | 0.22 |
| 1995 | 1.76 | 0.44 | 0.56 | 0.23 | 0.31 | 0.22 |
| 1996 | 1.72 | 0.43 | 0.55 | 0.23 | 0.30 | 0.21 |
| 1997 | 1.76 | 0.43 | 0.54 | 0.24 | 0.32 | 0.23 |
| 1998 | 1.70 | 0.42 | 0.55 | 0.22 | 0.29 | 0.21 |
| 1999 | 1.66 | 0.41 | 0.54 | 0.22 | 0.29 | 0.20 |
| 2000 | 1.66 | 0.40 | 0.54 | 0.22 | 0.29 | 0.20 |
| 2001 | 1.61 | 0.40 | 0.52 | 0.22 | 0.27 | 0.19 |
| 2002 | 1.56 | 0.39 | 0.51 | 0.21 | 0.27 | 0.19 |
| 2003 | 1.58 | 0.40 | 0.51 | 0.21 | 0.27 | 0.20 |
| 2004 | 1.55 | 0.38 | 0.50 | 0.20 | 0.27 | 0.20 |
| 2005 | 1.42 | 0.37 | 0.47 | 0.18 | 0.23 | 0.17 |
| 2006 | 1.36 | 0.34 | 0.46 | 0.19 | 0.22 | 0.16 |
| 2007 | 1.37 | 0.36 | 0.47 | 0.17 | 0.21 | 0.15 |
| 2008 | 1.36 | 0.35 | 0.45 | 0.18 | 0.22 | 0.16 |
| 2009 | 1.33 | 0.34 | 0.44 | 0.18 | 0.22 | 0.15 |
| 2010 | 1.28 | 0.33 | 0.42 | 0.16 | 0.21 | 0.16 |
| 2011 | 1.26 | 0.32 | 0.40 | 0.14 | 0.22 | 0.16 |
| 2012 | 1.36 | 0.35 | 0.45 | 0.17 | 0.22 | 0.16 |
| 2013 | 1.38 | 0.36 | 0.46 | 0.18 | 0.22 | 0.16 |
| 2014 | 1.36 | 0.36 | 0.43 | 0.17 | 0.23 | 0.16 |
| 2015 | 1.38 | 0.35 | 0.45 | 0.18 | 0.23 | 0.17 |
| 2016 | 1.26 | 0.33 | 0.43 | 0.17 | 0.20 | 0.14 |
| Total | **50.83** | **12.65** | **16.37** | **6.75** | **8.81** | **6.25** |

**Table S2.18. Manitoba Annual Emission from Fuel on Farm for Crop Production 1985-2016 (provincial and soil zone levels)**

| Year | Manitoba aggerate emission from fuel used in crop production (Mt CO_2_eq) | Manitoba emission from fuel used in crop production by soil zone (Mt CO_2_eq) | | | |  |
| --- | --- | --- | --- | --- | --- | --- |
|  |  | Dark Brown | Thin Black | Thick Black | Gray | |
| 1985 | 0.504 | 0.005 | 0.206 | 0.261 | 0.032 | |
| 1986 | 0.488 | 0.004 | 0.202 | 0.253 | 0.029 | |
| 1987 | 0.485 | 0.004 | 0.200 | 0.252 | 0.029 | |
| 1988 | 0.490 | 0.004 | 0.202 | 0.255 | 0.029 | |
| 1989 | 0.497 | 0.004 | 0.204 | 0.259 | 0.030 | |
| 1990 | 0.480 | 0.004 | 0.197 | 0.250 | 0.029 | |
| 1991 | 0.468 | 0.004 | 0.194 | 0.242 | 0.028 | |
| 1992 | 0.463 | 0.004 | 0.197 | 0.237 | 0.025 | |
| 1993 | 0.469 | 0.004 | 0.194 | 0.244 | 0.027 | |
| 1994 | 0.459 | 0.004 | 0.185 | 0.243 | 0.027 | |
| 1995 | 0.448 | 0.004 | 0.175 | 0.243 | 0.026 | |
| 1996 | 0.454 | 0.004 | 0.180 | 0.243 | 0.027 | |
| 1997 | 0.446 | 0.004 | 0.175 | 0.241 | 0.026 | |
| 1998 | 0.436 | 0.004 | 0.173 | 0.233 | 0.026 | |
| 1999 | 0.404 | 0.003 | 0.161 | 0.217 | 0.023 | |
| 2000 | 0.436 | 0.004 | 0.172 | 0.234 | 0.026 | |
| 2001 | 0.416 | 0.003 | 0.161 | 0.226 | 0.025 | |
| 2002 | 0.407 | 0.003 | 0.158 | 0.221 | 0.024 | |
| 2003 | 0.401 | 0.004 | 0.161 | 0.213 | 0.024 | |
| 2004 | 0.379 | 0.003 | 0.153 | 0.203 | 0.021 | |
| 2005 | 0.367 | 0.003 | 0.147 | 0.196 | 0.021 | |
| 2006 | 0.372 | 0.002 | 0.145 | 0.204 | 0.020 | |
| 2007 | 0.369 | 0.003 | 0.144 | 0.199 | 0.022 | |
| 2008 | 0.368 | 0.003 | 0.142 | 0.200 | 0.022 | |
| 2009 | 0.358 | 0.003 | 0.140 | 0.195 | 0.020 | |
| 2010 | 0.356 | 0.003 | 0.138 | 0.193 | 0.022 | |
| 2011 | 0.318 | 0.003 | 0.113 | 0.182 | 0.021 | |
| 2012 | 0.364 | 0.003 | 0.141 | 0.197 | 0.022 | |
| 2013 | 0.374 | 0.003 | 0.145 | 0.203 | 0.023 | |
| 2014 | 0.363 | 0.003 | 0.137 | 0.201 | 0.022 | |
| 2015 | 0.373 | 0.003 | 0.144 | 0.201 | 0.025 | |
| 2016 | 0.376 | 0.003 | 0.145 | 0.205 | 0.023 | |
| Total | **13.388** | **0.112** | **5.333** | **7.148** | **0.795** | |

**Table S2.19. Alberta Annual Emission from Fuel in Transportation 1985-2016 (provincial and soil zone levels)**

| Year | Alberta aggerate emission from fuel used in transportation (Mt CO_2_eq) | Alberta emission from fuel used in transportation by soil zone (Mt CO_2_eq) | | | | |
| --- | --- | --- | --- | --- | --- | --- |
|  |  | Brown | Dark Brown | Thin Black | Thick Black | Gray |
| 1985 | 0.202 | 0.021 | 0.055 | 0.040 | 0.038 | 0.048 |
| 1986 | 0.353 | 0.044 | 0.100 | 0.077 | 0.060 | 0.072 |
| 1987 | 0.325 | 0.040 | 0.090 | 0.068 | 0.055 | 0.073 |
| 1988 | 0.316 | 0.031 | 0.083 | 0.065 | 0.058 | 0.079 |
| 1989 | 0.513 | 0.063 | 0.140 | 0.108 | 0.084 | 0.117 |
| 1990 | 0.512 | 0.061 | 0.136 | 0.107 | 0.085 | 0.122 |
| 1991 | 0.510 | 0.070 | 0.140 | 0.105 | 0.082 | 0.114 |
| 1992 | 0.483 | 0.064 | 0.135 | 0.099 | 0.078 | 0.107 |
| 1993 | 0.538 | 0.070 | 0.152 | 0.113 | 0.087 | 0.116 |
| 1994 | 0.506 | 0.064 | 0.144 | 0.106 | 0.082 | 0.110 |
| 1995 | 0.644 | 0.087 | 0.183 | 0.130 | 0.103 | 0.142 |
| 1996 | 0.887 | 0.116 | 0.259 | 0.180 | 0.137 | 0.194 |
| 1997 | 0.858 | 0.117 | 0.261 | 0.187 | 0.136 | 0.157 |
| 1998 | 1.024 | 0.140 | 0.293 | 0.213 | 0.163 | 0.214 |
| 1999 | 1.071 | 0.149 | 0.312 | 0.225 | 0.162 | 0.223 |
| 2000 | 1.048 | 0.143 | 0.308 | 0.211 | 0.159 | 0.227 |
| 2001 | 0.925 | 0.120 | 0.271 | 0.194 | 0.139 | 0.200 |
| 2002 | 0.871 | 0.128 | 0.253 | 0.174 | 0.126 | 0.191 |
| 2003 | 0.979 | 0.142 | 0.284 | 0.192 | 0.147 | 0.213 |
| 2004 | 1.002 | 0.136 | 0.289 | 0.200 | 0.156 | 0.221 |
| 2005 | 1.004 | 0.140 | 0.296 | 0.199 | 0.152 | 0.218 |
| 2006 | 1.137 | 0.165 | 0.342 | 0.225 | 0.168 | 0.236 |
| 2007 | 1.114 | 0.159 | 0.337 | 0.216 | 0.174 | 0.228 |
| 2008 | 1.223 | 0.181 | 0.373 | 0.236 | 0.189 | 0.245 |
| 2009 | 1.157 | 0.155 | 0.339 | 0.226 | 0.181 | 0.257 |
| 2010 | 1.001 | 0.138 | 0.297 | 0.189 | 0.151 | 0.225 |
| 2011 | 1.292 | 0.163 | 0.369 | 0.289 | 0.190 | 0.281 |
| 2012 | 1.331 | 0.188 | 0.378 | 0.241 | 0.232 | 0.293 |
| 2013 | 1.322 | 0.195 | 0.377 | 0.254 | 0.197 | 0.300 |
| 2014 | 1.471 | 0.215 | 0.419 | 0.278 | 0.210 | 0.350 |
| 2015 | 1.487 | 0.217 | 0.421 | 0.280 | 0.214 | 0.356 |
| 2016 | 1.611 | 0.251 | 0.479 | 0.300 | 0.222 | 0.360 |
| Total | **28.720** | **3.972** | **8.310** | **5.730** | **4.421** | **6.287** |

**Table S2.20. Saskatchewan Annual Emission from Fuel in Transportation 1985-2016 (provincial and soil zone levels)**

| Year | Saskatchewan aggerate emission from fuel used in transportation (Mt CO_2_eq) | Saskatchewan emission from fuel used in transportation by soil zone (Mt CO_2_eq) | | | | |
| --- | --- | --- | --- | --- | --- | --- |
|  |  | Brown | Dark Brown | Thin Black | Thick Black | Gray |
| 1985 | 0.15 | 0.02 | 0.04 | 0.02 | 0.04 | 0.03 |
| 1986 | 0.20 | 0.04 | 0.07 | 0.03 | 0.03 | 0.02 |
| 1987 | 0.17 | 0.04 | 0.06 | 0.02 | 0.03 | 0.02 |
| 1988 | 0.27 | 0.05 | 0.08 | 0.04 | 0.06 | 0.04 |
| 1989 | 0.49 | 0.12 | 0.14 | 0.06 | 0.10 | 0.07 |
| 1990 | 0.57 | 0.12 | 0.18 | 0.08 | 0.11 | 0.08 |
| 1991 | 0.30 | 0.07 | 0.09 | 0.04 | 0.06 | 0.04 |
| 1992 | 0.27 | 0.06 | 0.09 | 0.04 | 0.05 | 0.03 |
| 1993 | 0.30 | 0.08 | 0.10 | 0.04 | 0.05 | 0.04 |
| 1994 | 0.42 | 0.10 | 0.13 | 0.06 | 0.08 | 0.06 |
| 1995 | 0.55 | 0.13 | 0.17 | 0.07 | 0.10 | 0.07 |
| 1996 | 0.79 | 0.18 | 0.25 | 0.10 | 0.15 | 0.11 |
| 1997 | 0.80 | 0.18 | 0.25 | 0.11 | 0.15 | 0.11 |
| 1998 | 0.59 | 0.13 | 0.19 | 0.08 | 0.11 | 0.08 |
| 1999 | 0.64 | 0.15 | 0.20 | 0.08 | 0.12 | 0.09 |
| 2000 | 0.65 | 0.15 | 0.20 | 0.09 | 0.12 | 0.09 |
| 2001 | 0.35 | 0.08 | 0.11 | 0.05 | 0.07 | 0.05 |
| 2002 | 0.29 | 0.07 | 0.09 | 0.05 | 0.05 | 0.03 |
| 2003 | 0.27 | 0.06 | 0.08 | 0.04 | 0.05 | 0.04 |
| 2004 | 0.32 | 0.08 | 0.10 | 0.04 | 0.06 | 0.04 |
| 2005 | 0.64 | 0.16 | 0.21 | 0.08 | 0.11 | 0.08 |
| 2006 | 0.71 | 0.16 | 0.24 | 0.10 | 0.12 | 0.09 |
| 2007 | 1.06 | 0.26 | 0.37 | 0.14 | 0.17 | 0.13 |
| 2008 | 1.17 | 0.27 | 0.38 | 0.16 | 0.21 | 0.15 |
| 2009 | 1.15 | 0.26 | 0.38 | 0.16 | 0.20 | 0.14 |
| 2010 | 1.31 | 0.35 | 0.45 | 0.16 | 0.20 | 0.15 |
| 2011 | 1.42 | 0.36 | 0.45 | 0.13 | 0.27 | 0.21 |
| 2012 | 1.55 | 0.39 | 0.51 | 0.20 | 0.25 | 0.19 |
| 2013 | 1.88 | 0.47 | 0.63 | 0.25 | 0.31 | 0.23 |
| 2014 | 2.53 | 0.67 | 0.79 | 0.30 | 0.45 | 0.33 |
| 2015 | 2.75 | 0.65 | 0.87 | 0.36 | 0.50 | 0.37 |
| 2016 | 2.65 | 0.68 | 0.87 | 0.35 | 0.43 | 0.31 |
| Total | **27.24** | **6.60** | **8.79** | **3.54** | **4.81** | **3.52** |

**Table S2.21. Manitoba Annual Emission from Fuel Used in Transportation 1985-2016 (provincial and soil zone levels)**

| Year | Manitoba aggerate emission from fuel Used in transportation (Mt CO_2_eq) | Manitoba emission from fuel used in transportation by soil zone (Mt CO_2_eq) | | | |  |
| --- | --- | --- | --- | --- | --- | --- |
|  |  | Dark Brown | Thin Black | Thick Black | Gray | |
| 1985 | 0.121 | 0.001 | 0.046 | 0.067 | 0.007 | |
| 1986 | 0.122 | 0.001 | 0.050 | 0.065 | 0.006 | |
| 1987 | 0.108 | 0.001 | 0.041 | 0.060 | 0.006 | |
| 1988 | 0.133 | 0.001 | 0.053 | 0.070 | 0.008 | |
| 1989 | 0.164 | 0.001 | 0.059 | 0.094 | 0.009 | |
| 1990 | 0.191 | 0.001 | 0.077 | 0.103 | 0.010 | |
| 1991 | 0.239 | 0.002 | 0.098 | 0.126 | 0.013 | |
| 1992 | 0.258 | 0.002 | 0.104 | 0.139 | 0.013 | |
| 1993 | 0.228 | 0.002 | 0.094 | 0.120 | 0.012 | |
| 1994 | 0.241 | 0.002 | 0.093 | 0.133 | 0.013 | |
| 1995 | 0.227 | 0.002 | 0.084 | 0.129 | 0.013 | |
| 1996 | 0.252 | 0.002 | 0.096 | 0.140 | 0.014 | |
| 1997 | 0.238 | 0.002 | 0.090 | 0.133 | 0.014 | |
| 1998 | 0.247 | 0.002 | 0.095 | 0.137 | 0.013 | |
| 1999 | 0.247 | 0.002 | 0.085 | 0.146 | 0.014 | |
| 2000 | 0.291 | 0.002 | 0.114 | 0.158 | 0.016 | |
| 2001 | 0.314 | 0.003 | 0.126 | 0.168 | 0.018 | |
| 2002 | 0.323 | 0.002 | 0.124 | 0.179 | 0.018 | |
| 2003 | 0.341 | 0.003 | 0.133 | 0.186 | 0.019 | |
| 2004 | 0.413 | 0.003 | 0.168 | 0.221 | 0.021 | |
| 2005 | 0.352 | 0.003 | 0.153 | 0.177 | 0.018 | |
| 2006 | 0.421 | 0.003 | 0.164 | 0.234 | 0.020 | |
| 2007 | 0.518 | 0.004 | 0.204 | 0.281 | 0.028 | |
| 2008 | 0.551 | 0.004 | 0.213 | 0.303 | 0.030 | |
| 2009 | 0.425 | 0.003 | 0.174 | 0.228 | 0.020 | |
| 2010 | 0.399 | 0.003 | 0.157 | 0.217 | 0.022 | |
| 2011 | 0.395 | 0.004 | 0.127 | 0.241 | 0.024 | |
| 2012 | 0.439 | 0.003 | 0.174 | 0.238 | 0.024 | |
| 2013 | 0.477 | 0.004 | 0.184 | 0.262 | 0.027 | |
| 2014 | 0.529 | 0.004 | 0.187 | 0.308 | 0.030 | |
| 2015 | 0.574 | 0.005 | 0.220 | 0.313 | 0.036 | |
| 2016 | 0.592 | 0.005 | 0.225 | 0.330 | 0.033 | |
| Total | 10.371 | 0.082 | 4.013 | 5.705 | 0.572 | |

**Table S2.22 Alberta Net GHG Balance and Value (1985–2016)**

| Year | Alberta net GHG balance (Mt CO_2_eq)  (parentheses indicate net sequestration or sink) | Alberta net GHG balance value (parentheses indicate debit value)  (millions in $2018)  CO_2_eq emitting price | | | |
| --- | --- | --- | --- | --- | --- |
|  |  | **$5** | **$10** | | **$15** |
| 1985 | 3.665 | (9.197) | | (18.394) | (27.591) |
| 1986 | 4.102 | (10.560) | | (21.121) | (31.681) |
| 1987 | 4.037 | (10.696) | | (21.392) | (32.088) |
| 1988 | 4.064 | (11.057) | | (22.115) | (33.172) |
| 1989 | 4.219 | (11.893) | | (23.785) | (35.678) |
| 1990 | 4.301 | (12.560) | | (25.120) | (37.681) |
| 1991 | 4.111 | (12.533) | | (25.065) | (37.598) |
| 1992 | 3.879 | (11.969) | | (23.938) | (35.907) |
| 1993 | 4.075 | (12.773) | | (25.546) | (38.319) |
| 1994 | 3.988 | (12.514) | | (25.027) | (37.541) |
| 1995 | 3.994 | (12.770) | | (25.540) | (38.310) |
| 1996 | 4.207 | (13.627) | | (27.255) | (40.882) |
| 1997 | 3.860 | (12.691) | | (25.383) | (38.074) |
| 1998 | 3.854 | (12.785) | | (25.570) | (38.355) |
| 1999 | 3.874 | (13.055) | | (26.110) | (39.164) |
| 2000 | 3.494 | (12.069) | | (24.138) | (36.208) |
| 2001 | 2.919 | (10.325) | | (20.651) | (30.976) |
| 2002 | 2.884 | (10.424) | | (20.849) | (31.273) |
| 2003 | 2.582 | (9.597) | | (19.194) | (28.791) |
| 2004 | 2.600 | (9.845) | | (19.689) | (29.534) |
| 2005 | 2.450 | (9.490) | | (18.979) | (28.469) |
| 2006 | 2.574 | (10.180) | | (20.360) | (30.541) |
| 2007 | 2.379 | (9.638) | | (19.275) | (28.913) |
| 2008 | 1.713 | (7.118) | | (14.236) | (21.354) |
| 2009 | 1.397 | (5.824) | | (11.647) | (17.471) |
| 2010 | 0.464 | (1.976) | | (3.953) | (5.929) |
| 2011 | 0.254 | (1.117) | | (2.233) | (3.350) |
| 2012 | 0.325 | (1.457) | | (2.914) | (4.370) |
| 2013 | (0.434) | 1.968 | | 3.935 | 5.903 |
| 2014 | 0.159 | (0.736) | | (1.471) | (2.207) |
| 2015 | 0.216 | (1.018) | | (2.035) | (3.053) |
| 2016 | (0.035) | 0.168 | | 0.335 | 0.503 |

**Table S2*.*23. Saskatchewan Net GHG Balance and Value (1985–2016)**

| Year | Net GHG balance (Mt CO_2_eq)  (parentheses indicate net sequestration or sink) | Net GHG balance value (parentheses indicate debit value)  (millions in $2018)  CO_2_eq emitting price | | |
| --- | --- | --- | --- | --- |
|  |  | **$5** | **$10** | **$15** |
| 1985 | 5.00 | (12.54) | (25.09) | (37.63) |
| 1986 | 5.30 | (13.64) | (27.28) | (40.92) |
| 1987 | 5.35 | (14.18) | (28.37) | (42.55) |
| 1988 | 4.74 | (12.89) | (25.79) | (38.68) |
| 1989 | 4.66 | (13.14) | (26.28) | (39.43) |
| 1990 | 5.40 | (15.76) | (31.52) | (47.27) |
| 1991 | 5.09 | (15.53) | (31.06) | (46.59) |
| 1992 | 4.81 | (14.85) | (29.70) | (44.55) |
| 1993 | 4.74 | (14.87) | (29.74) | (44.62) |
| 1994 | 4.93 | (15.46) | (30.91) | (46.37) |
| 1995 | 4.82 | (15.42) | (30.84) | (46.26) |
| 1996 | 4.97 | (16.11) | (32.23) | (48.34) |
| 1997 | 4.66 | (15.31) | (30.62) | (45.93) |
| 1998 | 3.31 | (10.98) | (21.96) | (32.94) |
| 1999 | 3.14 | (10.60) | (21.20) | (31.80) |
| 2000 | 2.74 | (9.47) | (18.94) | (28.40) |
| 2001 | 2.65 | (9.39) | (18.77) | (28.16) |
| 2002 | 2.42 | (8.75) | (17.49) | (26.24) |
| 2003 | 1.66 | (6.17) | (12.35) | (18.52) |
| 2004 | 1.19 | (4.51) | (9.02) | (13.53) |
| 2005 | 0.88 | (3.42) | (6.84) | (10.26) |
| 2006 | 0.63 | (2.51) | (5.02) | (7.53) |
| 2007 | 0.56 | (2.26) | (4.52) | (6.78) |
| 2008 | (0.39) | 1.62 | 3.24 | 4.85 |
| 2009 | (0.76) | 3.18 | 6.36 | 9.55 |
| 2010 | 0.97 | (4.13) | (8.25) | (12.38) |
| 2011 | 0.45 | (1.96) | (3.93) | (5.89) |
| 2012 | (0.16) | 0.73 | 1.46 | 2.20 |
| 2013 | (1.57) | 7.13 | 14.26 | 21.40 |
| 2014 | (0.00) | 0.00 | 0.01 | 0.01 |
| 2015 | (0.12) | 0.57 | 1.15 | 1.72 |
| 2016 | 0.12 | (0.60) | (1.19) | (1.79) |

**Table S2.24. Manitoba Net GHG Balance and Value (1985–2016)**

| Year | Manitoba net GHG balance (Mt CO_2_eq)  (parentheses indicate net sequestration or sink) | Manitoba net GHG balance value (parentheses indicate debit value)  (millions in $2018)  CO_2_eq emitting price | | |
| --- | --- | --- | --- | --- |
|  |  | **$5** | **$10** | **$15** |
| 1985 | 2.135 | (5.358) | (10.716) | (16.074) |
| 1986 | 2.133 | (5.492) | (10.984) | (16.476) |
| 1987 | 2.010 | (5.325) | (10.650) | (15.975) |
| 1988 | 1.910 | (5.196) | (10.392) | (15.588) |
| 1989 | 1.952 | (5.502) | (11.003) | (16.505) |
| 1990 | 2.122 | (6.197) | (12.395) | (18.592) |
| 1991 | 2.136 | (6.512) | (13.024) | (19.536) |
| 1992 | 2.144 | (6.615) | (13.231) | (19.846) |
| 1993 | 2.080 | (6.518) | (13.037) | (19.555) |
| 1994 | 2.150 | (6.747) | (13.494) | (20.240) |
| 1995 | 2.093 | (6.693) | (13.386) | (20.079) |
| 1996 | 2.168 | (7.021) | (14.042) | (21.062) |
| 1997 | 2.010 | (6.607) | (13.213) | (19.820) |
| 1998 | 1.935 | (6.418) | (12.836) | (19.254) |
| 1999 | 2.090 | (7.042) | (14.084) | (21.126) |
| 2000 | 2.039 | (7.042) | (14.085) | (21.127) |
| 2001 | 1.964 | (6.947) | (13.894) | (20.841) |
| 2002 | 1.921 | (6.946) | (13.891) | (20.837) |
| 2003 | 1.978 | (7.352) | (14.704) | (22.056) |
| 2004 | 1.978 | (7.492) | (14.985) | (22.477) |
| 2005 | 1.826 | (7.074) | (14.148) | (21.222) |
| 2006 | 1.849 | (7.312) | (14.624) | (21.935) |
| 2007 | 1.952 | (7.906) | (15.812) | (23.718) |
| 2008 | 1.942 | (8.069) | (16.138) | (24.207) |
| 2009 | 1.575 | (6.563) | (13.126) | (19.689) |
| 2010 | 1.713 | (7.288) | (14.577) | (21.865) |
| 2011 | 2.126 | (9.355) | (18.710) | (28.065) |
| 2012 | 1.504 | (6.738) | (13.476) | (20.214) |
| 2013 | 1.731 | (7.841) | (15.682) | (23.523) |
| 2014 | 2.061 | (9.559) | (19.118) | (28.678) |
| 2015 | 1.938 | (9.115) | (18.230) | (27.345) |
| 2016 | 2.103 | (10.067) | (20.134) | (30.201) |
